# Ameliorative effect of curcumin and/or cerium oxide nanoparticles on diabetes of rats: biochemical, molecular, and pathological insights

**DOI:** 10.1038/s41598-026-66612-z

**Published:** 2026-08-21

**Authors:** Ahmed A. Elzahby, Eman M. Abd El-maksoud, Omaima Ahmed, Magdy A. Ghoneim, Shaimaa Kamel

**Affiliations:** 1 Physiology and Biochemistry Department, Faculty of Veterinary Medicine, Ain Shams University, Cairo, Egypt; 2 Biochemistry Department, Faculty of Veterinary Medicine, Alexandria University, Alexandria, Egypt; 3 Cytology and Histology Department, Faculty of Veterinary Medicine, Cairo University, Giza, Egypt; 4 Department of Biochemistry and Molecular Biology, Faculty of Veterinary Medicine, Cairo University, Giza, Egypt

**Keywords:** Alloxan-induced diabetes, Nanocurcumin, Cerium oxide nanoparticles, Insulin signaling, Oxidative-antioxidative status, NF-κBp65, Biochemistry, Biotechnology, Diseases, Drug discovery

## Abstract

Diabetes mellitus encompasses a spectrum of metabolic dysfunctions fundamentally defined by a chronic state of hyperglycemia and oxidative cytotoxicity, where current therapies often fail to arrest progressive β-cell failure. This study evaluated the protective efficacy and mechanistic pathways of Nanocurcumin, Cerium Oxide Nanoparticles (CNPs), and their combinatorial formulation in alleviating pathologies in an alloxan-induced type 1 diabetic rat model. Nanoparticle characterization via transmission electron microscopy and XRD analysis confirmed the successful preparation of spheroidal Nanocurcumin (30 ± 5 nm) and CNPs (17 ± 2 nm). A total of fifty male albino rats were randomly allocated into five distinct experimental groups: negative control, diabetic (alloxan 150 mg/kg), Nanocurcumin (5 mg/kg), CNPs (45 mg/kg), and a combination group of both dosages. Intraperitoneal treatments were administered for three weeks pre-induction of diabetes and four weeks post-induction. Blood and tissue samples (liver and pancreas) were collected. The combination therapy demonstrated superior efficacy over monotherapies, reducing blood glucose by 69% and restoring serum insulin by 50%. It significantly ameliorated dyslipidemia, lowering total cholesterol by up to 50% and triglycerides by up to 56%, and attenuated hepatorenal injury, evidenced by reduced liver enzyme activities (ALT decreased by up to 36% and AST by up to 21%) alongside normalized urea and creatinine levels. In hepatic and pancreatic tissues, the treatments attenuated oxidative stress by reducing the lipid peroxidation marker MDA by 36–65% and enhancing antioxidant defenses, including increases in GSH (up to 58%), GPx (up to 25%), and catalase activities (up to 58%). Molecular analysis revealed upregulated hepatic IR, PI3K, and AKT expression, alongside suppression of pancreatic VEGF and iNOS. Histopathological examination confirmed islet architecture preservation and reduced NF-κB p65 immunoreactivity. Co-administration of Nanocurcumin and CNPs provides a potent, multi-targeted protective strategy, offering a promising preclinical avenue for diabetes management beyond traditional glycemic control, which warrants further translational investigation.

## Introduction

The epidemiological trajectory of diabetes mellitus (DM) indicates a rapidly escalating burden on global healthcare systems. Characterized by chronic hyperglycemia, the global prevalence of diabetes currently affects approximately 463 million individuals, a figure projected to surge to 700 million within the next 25 years^1^. Consequently, advancing our pathophysiological understanding and identifying novel therapeutic interventions for this widespread metabolic disorder is of critical scientific significance.

Clinically, diabetes is classified into three primary etiologies: type 1, type 2, and gestational diabetes mellitus. Type 1 diabetes is driven by the autoimmune-mediated destruction of endogenous insulin-secreting pancreatic β-cells, predominantly presenting in juvenile populations^2^. Conversely, Type 2 diabetes stems from a progressive onset of peripheral insulin resistance coupled with a subsequent decline in pancreatic insulin secretion, a pathogenesis strongly correlated with chronic dietary and lifestyle factors in adults^3^. Gestational diabetes shares similar underlying mechanisms but is restricted to pregnancy, notably increasing the future risk of hyperglycemia for offspring born to affected mothers^2,3^.

While various factors, such as anomalous insulin secretion, antagonist molecules, and genetic alterations in the insulin receptor (IR) or its substrates, have been hypothesized, current consensus establishes that impaired post-receptor signaling pathways are the fundamental drivers of insulin resistance within target tissues. Furthermore, this impairment is linked to the reduced expression, accelerated breakdown, or deficient tyrosine phosphorylation of initial insulin signaling molecules. Specifically, the abnormal phosphorylation of serine or threonine residues on IRS proteins diminishes their functional capacity, effectively obstructing subsequent intracellular pathways^4^.

Diabetes mellitus is frequently accompanied by an overproduction of free radicals and a compromised antioxidant system. While baseline oxidative stress facilitates certain physiological functions like glucose-induced insulin release, excessive oxidative levels provoke severe biological damage when oxidizing agents overwhelm the body's endogenous antioxidant defenses. The accumulation of reactive oxygen species (ROS), including hydrogen peroxide (H₂O₂) and hydroxyl radicals, significantly intensifies this oxidative burden^4^.

To quantify this stress, byproducts such as malondialdehyde (MDA) and glutathione disulfide (GSSG) serve as reliable biomarkers^1^. Conversely, endogenous antioxidants, including reduced glutathione (GSH), alongside essential enzymes like glutathione peroxidase (GPx), superoxide dismutase (SOD), and catalase (CAT), exert significant protective effects against diabetic pathology^5^.Evaluating total antioxidant capacity (TAC) and total thiol molecules (TTM) is also critical, given the structural reliance of many antioxidant mechanisms on thiol groups^1,5^.

To counteract this oxidative damage, recent research has explored the therapeutic potential of organic and inorganic nanoparticle antioxidants^6^. Cerium oxide nanoparticles (CNPs), synthesized from the lanthanide group of rare-earth metals, have demonstrated substantial anti-inflammatory and antidiabetic properties^7^. Pharmacologically, the antidiabetic efficacy of CNPs is largely attributed to their ability to attenuate pancreatic β-cell apoptosis. This potent radical-scavenging action stems from their unique capacity to undergo reversible redox cycling, seamlessly transitioning between the Ce^3^⁺ and Ce^4^⁺ oxidation states in direct response to the local concentration of ROS^8^. Specifically, CNPs in the Ce^3^⁺ state neutralize ROS to generate Ce^4^⁺, effectively mimicking SOD activity, while in the Ce^4^⁺ state, they catalyze the breakdown of peroxides to yield Ce^3^⁺, water, and oxygen, functioning analogously to catalase. This continuous, auto-regenerative cycling fundamentally drives the compound's robust antioxidant capacity. Furthermore, the administration of CNPs has been shown to mitigate diabetes-induced systemic weight loss, restoring normal growth trajectories comparable to non-diabetic subjects^7^.

Similarly, curcumin, the chief bioactive phytochemical extracted from the rhizomes of *Curcuma longa*, has surfaced as a viable investigational therapeutic strategy for diabetes management in experimental models due to its inherent antioxidant and anti-inflammatory characteristics^9^. Despite its limited hydrophilicity at non-alkaline pH levels, advancements in nanotechnology have significantly enhanced its bioavailability and systemic delivery^9,10^. Curcumin nanoformulations are now extensively investigated as safe, cost-effective interventions in experimental diabetic models, demonstrating the capacity to mitigate weight loss, decrease circulating blood glucose, and lower glycosylated hemoglobin (HbA1c) concentrations^10,11^. Mechanistically, nanoparticulate curcumin exhibits substantial potential in reversing pancreatic β-cell dysfunction by suppressing poly (ADP-ribose) polymerase-1 (PARP-1) activation and regulating the nuclear translocation of nuclear factor kappa-light-chain-enhancer of activated B cells (NF-κB), which is typically provoked by pro-inflammatory cytokines^12^. This targeted molecular interference enhances islet survival, curbs intracellular ROS buildup, elevates the operational capacity of endogenous antioxidant enzymes, and bolsters glutathione concentrations, thereby shielding insular cells from oxidative damage^13^.

This research aims to measure the impact of both curcumin and/or cerium oxide nanoparticle injection on the diabetic status of rats. Additionally, it illustrates some of the pathophysiological pathways driving oxidative damage and inflammatory responses in diabetes mellitus.

## Materials and methods

### Experimental animals

A total experiment comprising fifty male Wistar albino rats (5-week-old/100-120 g) was procured from the Research Institute of Ophthalmology (Giza, Egypt). The animals were maintained in a climate-controlled animal facility with a regulated ambient temperature of 22 ± 2 °C, a relative humidity of 50–60%, and a standardized 12-h light/12-h dark cycle. They were provided with water ad libitum and fed a standardized laboratory rodent chow (manufactured by El-Nasr Chemical Company, Egypt). The nutritional composition of this basal diet was formulated to contain approximately 20–22% crude protein, 3–5% crude fat, and 4–6% crude fiber, supplemented with essential minerals and vitamin complexes to meet the standard physiological requirements for growing male albino rats. All experiments adhered strictly to the ethical protocols established by the Animal Bioethics Committee at the Faculty of Veterinary Medicine, Cairo University.

### Chemicals

Alloxan, Cerium oxide nanoparticles, and Curcumin nanoparticles were purchased from Nanogate Company, Egypt. Nanoparticles were prepared and characterized by the supplying agent as follows.

### Preparation of cerium oxide nanoparticles

#### Materials

High-purity cerium nitrate (Ce(NO₃)₃0.6H₂O) and hexamethylenetetramine (HMT) were sourced from Loba Chemie (India). Concurrently, sodium hydroxide (NaOH) was procured through Millipore Merck (Germany). All precursor reagents were utilized in their as-received condition, requiring no subsequent refinement or purification steps.

#### Preparation methods

The generation of nanoparticulate cerium oxide was achieved through a targeted hydrothermal synthesis protocol^14^. In brief, the synthesis protocol commenced with the aqueous dissolution of 1.5 g of Ce(NO₃)₃·6H₂O, immediately followed by the swift incorporation of a NaOH solution (16 g) under continuous mechanical agitation. This reactive mixture was allowed to equilibrate for 30 min before being sealed within a 50 mL autoclave. Hydrothermal processing was subsequently executed at 200 °C for a duration of 24 h. Upon natural cooling to ambient conditions, the solid phase was isolated via centrifugation. The recovered pellets underwent successive washing cycles and were desiccated overnight at 60 °C, ultimately yielding a fine powder of ceria nanoparticles characterized by a dimensional range of 20–25 nm.

#### Characterization techniques

Transmission electron microscope (TEM)

To ascertain the morphological characteristics and dimensional parameters of the prepared nanomaterials, high-resolution transmission electron microscopy (HR-TEM) was conducted utilizing a Thermo-Scientific Talos F200i instrument operated at a 200 kV accelerating voltage. Specimen preparation for this microstructural analysis involved the precise deposition of a colloidal suspension droplet onto a 300-mesh copper grid coated with Formvar and carbon (Ted Pella). Following application, the respective solvent was permitted to passively volatilize under ambient atmospheric conditions prior to imaging^14^.

XRD Analysis

To evaluate the crystallographic structure of the synthesized material, X-ray diffraction (XRD) measurements were executed utilizing an XPERT-PRO powder diffractometer. The analytical scans encompassed a 2θ angular range from 20° to 80°, operating with a highly resolved step increment of 0.001° and an incident radiation wavelength (Kα) of 1.54614 Å^14^.

### Preparation of curcumin nanopowder

Curcumin nanopowder was prepared via a ball milling approach^15,16^. Briefly, curcumin powder (Loba Chemie, India) underwent mechanical milling in a planetary ball mill (PM 400) for a total duration of 10 h at 350 rpm, incorporating 3-min pause intervals.

#### Characterization techniques

Morphological and dimensional evaluations of the nanoparticles were conducted utilizing a Talos F200i high-resolution transmission electron microscope (Thermo-Scientific), operating at an accelerating voltage of 200 kV. To facilitate microstructural imaging, specimens were prepared by precisely depositing a droplet of the colloidal suspension onto a 300-mesh, formvar/carbon-coated copper grid (Ted Pella). The solvent was subsequently permitted to volatilize passively under ambient atmospheric conditions. Finally, quantitative assessments of the mean particle diameter and overall size distribution were derived through the application of dedicated image analysis software.

### Preparation of curcumin and cerium oxide nanoparticles stock

Both nanocurcumin and CNPs were freshly dissolved in phosphate-buffered saline (PBS; pH 7.4) containing 10% analytical-grade dimethyl sulfoxide (DMSO; Sigma-Aldrich, Germany, analytical grade) to ensure optimal dispersion prior to administration^17^.

### Experimental design and animal grouping

Following 2 weeks of acclimatization, the experimental animals were randomly allocated into five equivalently sized groups:

1. G1 (Control Negative): Untreated group received phosphate-buffered saline (PBS) with 10% dimethyl sulfoxide (DMSO).
2. G2 (Diabetic): It received alloxan (150 mg/kg/i.p.).
3. G3 (Nanocurcumin): It received nanocurcumin (5 mg/kg/i.p.) (3 times/week) for 3 weeks before establishing the experimental diabetic model and for another 4 weeks after the induction of diabetes.
4. G4 (CNPs): It received CNPs (45 mg/kg/i.p.) (3 times/week) for 3 weeks before establishing the experimental diabetic model and for another 4 weeks after the induction of diabetes.
5. G5 (Combination of nanocurcumin and CNPs): It received both nanocurcumin (5 mg/kg/i.p.) and CNPs (45 mg/kg/i.p.) (3 times/week) for 3 weeks before establishing the experimental diabetic model and for another 4 weeks after the induction of diabetes.

The selection of therapeutic dosages for this study was established based on previous in vivo research demonstrating their safety, antidiabetic efficacy, and antioxidative potential. Specifically, the dosage of 5 mg/kg/i.p. for nanocurcumin was adopted based on prior findings, which proved this concentration safely prevents streptozotocin-induced inflammation and apoptosis in pancreatic beta cells^18^. Similarly, the dose of 45 mg/kg/i.p. for cerium oxide nanoparticles (CNPs) was selected based on an established experimental diabetic rat model that confirmed its potent antiapoptotic and antioxidative effects without inducing toxicity^19^.

### Induction of type 1 diabetes

After 3 weeks of receiving phosphate buffer saline (PBS) with 10% dimethyl sulfoxide (DMSO), Group 2 (G2) subjects were injected intraperitoneally with alloxan monohydrate (150 mg/kg) prepared in a distilled water vehicle immediately before use, after 12 h of fasting. On the third day of alloxan injection, blood glucose was determined, and rats with glucose levels more than 200 mg/dl were classified as diabetic^20^. Animals with blood glucose levels lower than 200 mg/dl received a second dosage of alloxan (50 mg/kg/i.p.) to induce type 1 diabetes in these animals.

Animals in G3, 4, and 5 followed the same pattern for induction of diabetes as G2 animals, but after 3 weeks of administration with nanocurcumin, cerium oxide nanoparticles, or both, alternatively.

### Sample collection

Upon conclusion of the study, the subjects underwent a 12-h overnight fast prior to sedation with ketamine (60 mg/kg) and xylazine (5 mg/kg). Blood samples were collected by cardiac puncture in fluoride, serum, and EDTA tubes. Serum was isolated via centrifugation at 4000 rpm for 10 min, which was then aliquoted into microcentrifuge tubes and stored at − 20 °C for subsequent biochemical assays^21^. Fluoride-collecting tubes were also centrifuged at 4000 rpm for 10 min, then blood glucose was measured directly. While the EDTA tubes were used for assaying complete blood count (CBC) from whole blood samples. Also, the liver and pancreas were sectioned into three distinct portions. The first part was homogenized in 1 × PBS (pH 7.4) and centrifuged at 12,000 rpm for 15 min to prepare a 10% tissue homogenate for the determination of oxidative stress and antioxidant biomarkers^22^. About 0.1 g of the liver and pancreatic tissues were kept in RNA extraction buffer containing RNase inhibitor and used for gene expression analysis. The final segment was promptly immersed in 10% neutral buffered formalin (NBF) for histological preservation for histology and immunohistochemistry.

### Blood biochemical analysis

#### Blood glucose level

Blood glucose was measured in plasma samples by using a commercial kit from Spectrum Diagnostics, Egypt Company for Biotechnology, using a UV-2100 spectrophotometer^23^.

#### Hormonal analysis

Measurement of serum insulin was performed via a rat-specific ELISA kit (CAT. NO. 201-11-0708) (Shanghai Sunred Biological Technology Co., China). Meanwhile, total T3 and T4 levels were assayed on the IMMULITE 2000 platform (Siemens).

#### Total cholesterol (TC) and triglycerides (TAG)

Circulating concentrations of total cholesterol (TC) and triacylglycerols (TAG) were quantified in serum specimens utilizing commercial colorimetric assays sourced from Spectrum Diagnostics (Egyptian Company for Biotechnology). All analytical protocols were executed in strict adherence to the manufacturer’s standardized instructions, with absorbance measurements recorded via a UV 2100 spectrophotometer^24^.

#### Liver function tests

Serum activities of aspartate aminotransferase (AST) and alanine aminotransferase (ALT) were quantified using commercial colorimetric kits (Spectrum Diagnostics) in accordance with the manufacturer's protocol (Egyptian Company for Biotechnology). All quantification procedures were executed in precise alignment with the manufacturer’s established protocols, utilizing a UV 2100 spectrophotometer for absorbance data collection^25^.

#### Kidney function tests

Serum concentrations of creatinine and urea were measured with colorimetric kits from Spectrum Diagnostics, following the manufacturer's protocol (Egyptian Company for Biotechnology). These diagnostic evaluations were performed in strict compliance with the manufacturer’s standardized directives, with all optical density measurements recorded via a UV 2100 spectrophotometer.

#### Hematological analysis

CBC was performed using an automated veterinary hematology analyzer (Sysmex)^26^.

#### Determination of some oxidative stress and antioxidant biomarkers

Malondialdehyde (MDA) levels were quantified in 10% homogenates of liver and pancreatic tissues, following the method described by^27^, reduced glutathione (GSH) according to^28^, Glutathione peroxidase activity^29^, and catalase enzyme activity^30^.

#### qRT-PCR analysis for genes in hepatic and pancreatic tissues

To evaluate gene expression, the mRNA levels of hepatic PI3K, AKT, and IR, alongside pancreatic VEGF and iNOS, were quantified via qRT-PCR. Values were normalized to the β-actin gene, which served as the internal housekeeping control. Total RNA was extracted from approximately 100 mg of pancreatic and hepatic tissue samples using a specialized isolation kit (Applied Biotechnology, Egypt) according to the manufacturer's protocol^31^. Following the assessment of RNA concentration and purity via NanoDrop spectrophotometry^32^, the samples underwent reverse transcription using the ABT H-minus cDNA synthesis kit (Applied Biotechnology, Egypt)^33^. The quantification of cDNA was executed via real-time PCR utilizing the ABT 2X SYBR GREEN Master Mix (Applied Biotechnology), strictly adhering to the protocols outlined by the manufacturer^34^. The NCBI Primer-BLAST utility (https://www.ncbi.nlm.nih.gov/tools/primer-blast/) was employed to design all oligonucleotide primers used in this study; the resulting sequences are detailed in Table 1. The thermocycling protocol commenced with an initial denaturation step at 95 °C for 3 min. This was followed by 45 amplification cycles consisting of denaturation at 95 °C for 15 s, annealing at 60 °C for 30 s, and extension at 72 °C for 30 s^35^. Negative controls were used^23^, and each qRT-PCR was performed in triplicate^36^**.** Relative gene expression was quantified utilizing the comparative 2^−ΔΔCT^ method^37,38^.

**Table 1:** Primer sequences used for qRT-PCR.

Gene symbol	Gene description	Accession number	Primer sequence
IR^39^	Insulin Receptor	NM_017071.2	F: 5′-TCTTCTACAGCGAGGAGAAC-3′ R: 5′-TCATAGGTCCGTTTGATGCT-3′
AKT	Protein Kinase B	NM_033230.3	F: 5′‐CTGCCCTTCTACAACCAGGA‐3′ R: 5′‐GTGCTGCATGATCTCCTTGG‐3′
PI3K	Phosphatidylinositol 3-kinase	NM_133399.3	F: 5′‐ TAGTGTCCGGGAAAATGGCT ‐3′ R: 5′‐ GGCATGCTCTTCGATCACAG ‐3′
iNOS	Inducible Nitric Oxide Synthase	NM_012611.3	F: 5′-GGTGAGGGGACTGGACTTTTAG-3′ R: 5′-TTGTTGGGCTGGGAATAGCA-3′
VEGF	Vascular endothelial growth factor	NM_001287110.1	F: 5′- TTCCTGTAGACACACCCACC ‐3′ R: 5′- TCCTCCCAACTCAAGTCCAC ‐3′
β-actin^39^	Beta-actin	NM_031144.3	F: 5′-TCAAGATCATTGCTCCTCCT-3′ R: 5′-GTGTAAAACGCAGCTCAGTA-3′

#### Histopathology examination

##### Light microscopy

Following harvesting, liver and pancreatic specimens from all five cohorts were promptly immobilized in 10% neutral buffered formalin (NBF) for a 72-h period. The fixed tissues underwent subsequent dehydration through a graded series of ethanolic solutions, followed by xylene-mediated clearing and inclusion in paraffin blocks. Histopathological sections: the tissue blocks were sectioned at a thickness of 3–4 μm and subsequently stained with hematoxylin and eosin (H&E) in accordance with the protocols established by^40^. Representative micrographs were then acquired across varying magnifications utilizing a Leica microscope (Leica Microsystems, Switzerland). Histopathological lesions were scored using a semi-quantitative scoring system. In the pancreatic islet lesions, it was scored by a three-point scoring system (0 = absent, 1 = mild, 2 = moderate, 3 = severe) according to the degree of presence of it. In the hepatic lesions, according to^41^. Necrosis and inflammatory changes were scored on a scale reflecting the severity (0 = absent; 1 = mild; 2 = moderate; 3 = marked), relied on the extent and distribution of the lesions observed across multiple microscopic fields.

##### Immunohistochemical examination of NF-κBp65

Immunohistochemistry was carried out on deparaffinized pancreatic and hepatic sections (4-μm thick) for detection of inflammation according to the manufacturer’s protocol. Sections were immersed in 0.3% hydrogen peroxide (H_2_O_2_) in PBS, pH 7, for 15 min for deactivation of endogenous peroxidase. Then, probed with rabbit polyclonal antibodies (unconjugated) (PA-527617, Thermo Fisher Scientific) diluted in BSA-PBS 1:100 for 1 h, then washed thoroughly with PBS. After that, tissue sections were incubated with secondary antibody, Horseradish peroxidase (HRP) Envision Kit (DAKO), washed with PBS, and incubated for 10–15 min with 3,3'-diaminobenzidine (DAB). Sections were washed, counterstained in hematoxylin, dehydrated in ethanol, cleared with xylene, and covered with a coverslip for microscopic examination. Nuclear Factor kappa light chain enhancer of activated B cells p65 (NF-κBp65)-positive sections were brown colored.

##### Image analysis of immunohistochemical sections

The positive sections were assessed using Leica Qwin 500 software (Leica Microsystems, Switzerland) for morphological analysis. NF-κBp65 immunostaining was quantified as area percentage in different slides (n = 5 fields/group) at magnification power × 400. The areas displaying brown color immunoreaction (positive) were selected for estimation. Mean values and standard error of the mean (SEM) of each specimen were obtained and statistically analyzed^42^.

#### Statistical analysis

Numerical data obtained throughout the study are presented as the mean ± SEM. To evaluate the statistical differences across the various experimental groups, Statistical evaluations were performed via a one-way analysis of variance (ANOVA) utilizing the IBM SPSS Statistics suite (Version 27.0). Where significant variance was identified, specific inter-group comparisons were further refined. Following the initial variance analysis, Tukey's post hoc comparisons were applied. Across all evaluations, statistical significance was defined by a *p*-value of less than 0.05.

## Results

### Characterization of cerium oxide nanoparticles (CNPs)

**Fig. 1:**
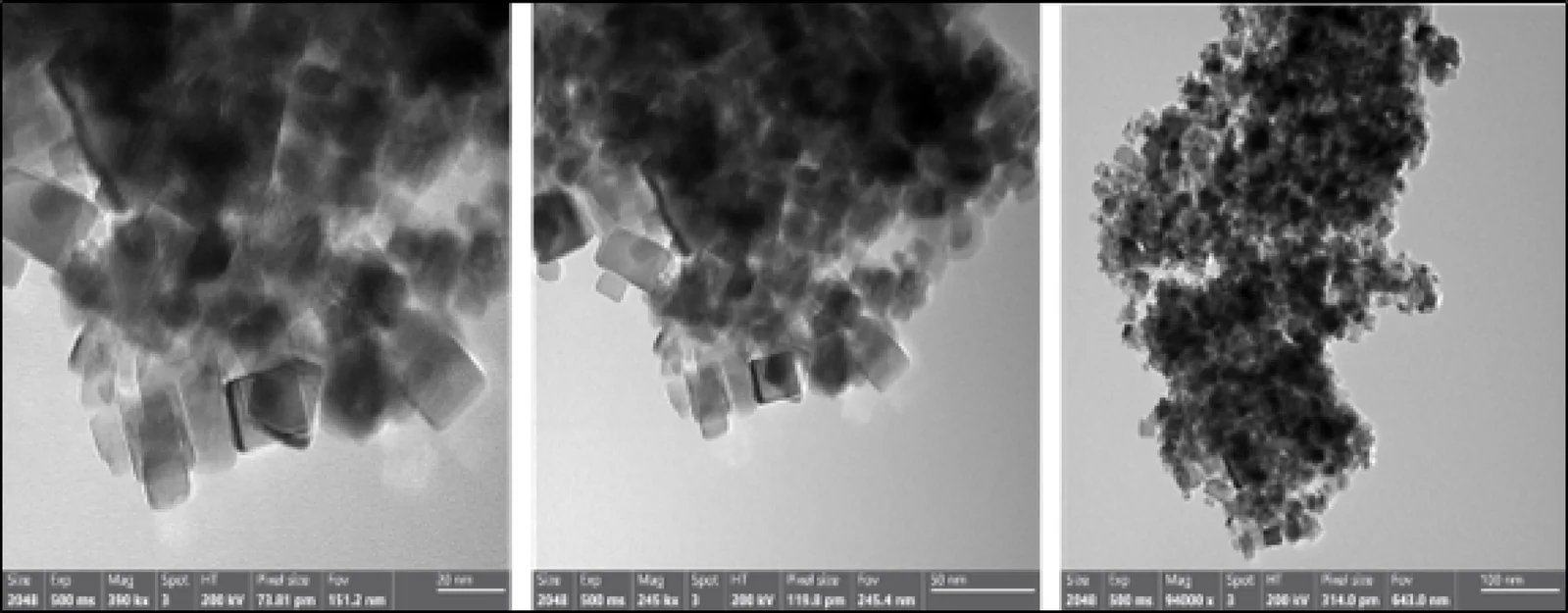
The TEM images of CNPs (size 17 ± 2 nm).

**Fig. 2:**
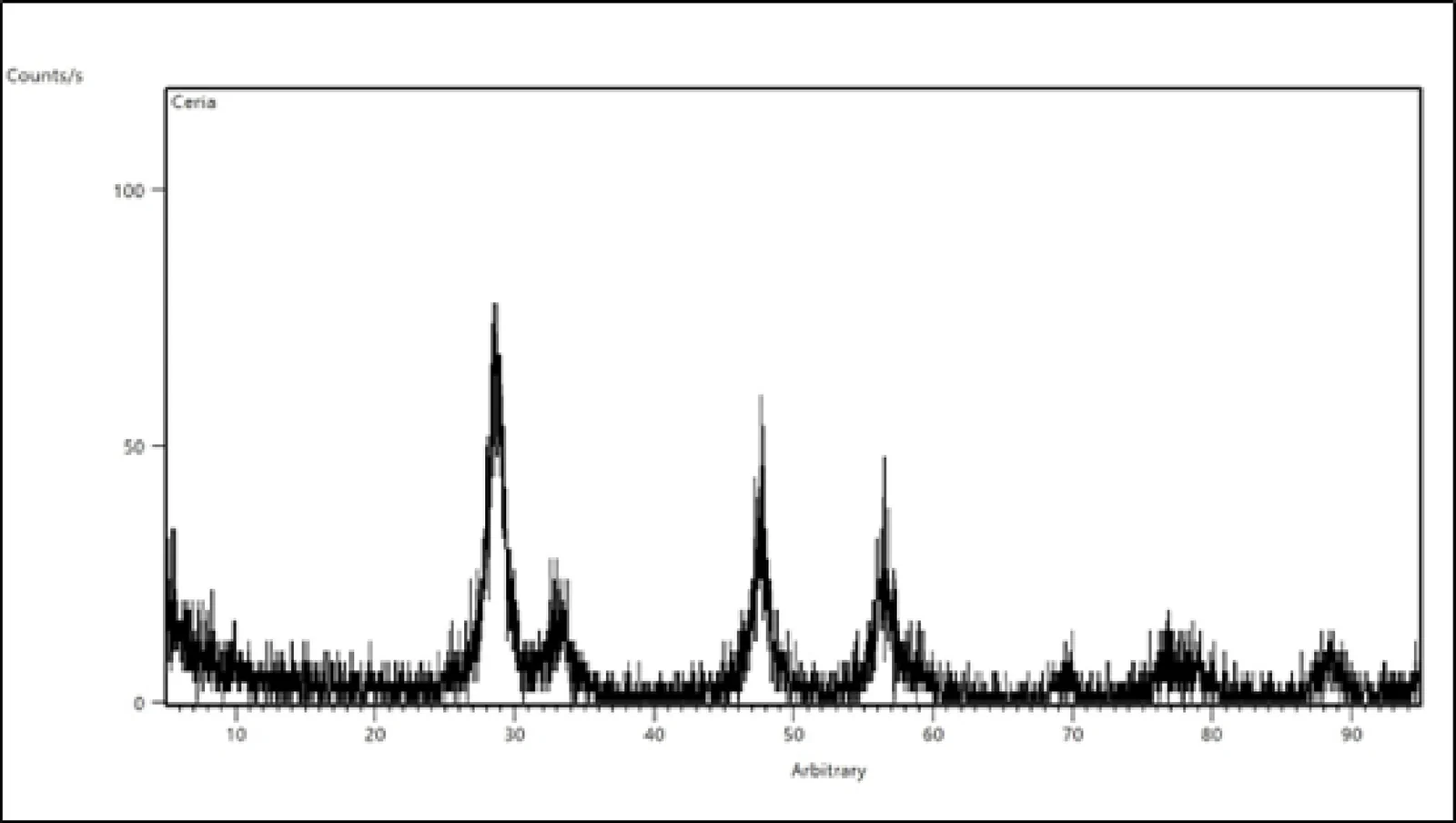
The XRD pattern of CNPs.

The average size of the CNPs (17 ± 2 nm) was calculated from the acquired TEM micrographs using an image analysis software package (Fig. 1). This ultra-small nanoscale dimension is highly relevant to our therapeutic study design, as it maximizes the surface-area-to-volume ratio, thereby increasing the exposed catalytic sites required for efficient reactive oxygen species scavenging. Furthermore, X-ray diffraction (XRD) patterns were acquired utilizing the XPERT-PRO Powder Diffractometer system, as shown in Fig. 2. This confirmed the specific crystalline structure of the synthesized material, which is essential for facilitating the auto-regenerative Ce^3^⁺/Ce^4^⁺ redox cycling that underlies its potent antioxidant efficacy in diabetic tissues^14^.

### Characterization of curcumin nanoparticles

**Fig. 3:**
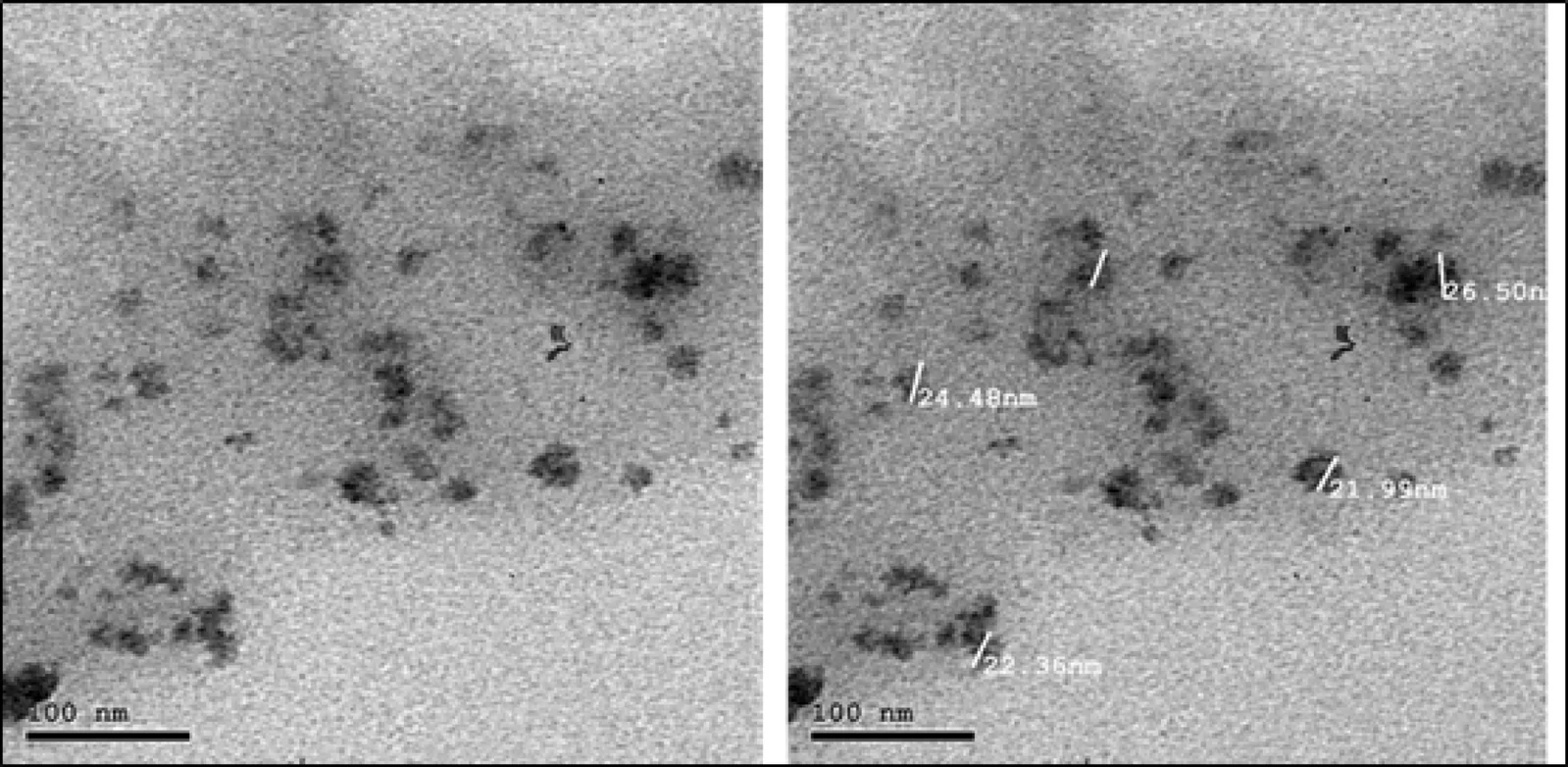
The TEM images of the prepared curcumin nano-emulsion.

The general properties of Curcumin Nanoparticles were examined by TEM, with size distribution and average size determined using an image analysis software package, which showed its powder form with an average size of 30 ± 5 nm and a spheroidal shape (Fig. 3). This specific morphological profile and ultra-small size are directly relevant to our in vivo study design; reducing bulk curcumin to this nanoscale significantly enhances its aqueous dispersion and systemic bioavailability, thereby overcoming its inherent hydrophobicity and enabling targeted cellular delivery within the diabetic model^15,16^.

### Effects of nanocurcumin and/or cerium oxide nanoparticles (CNPs) on glycemic level

**Fig. 4:**
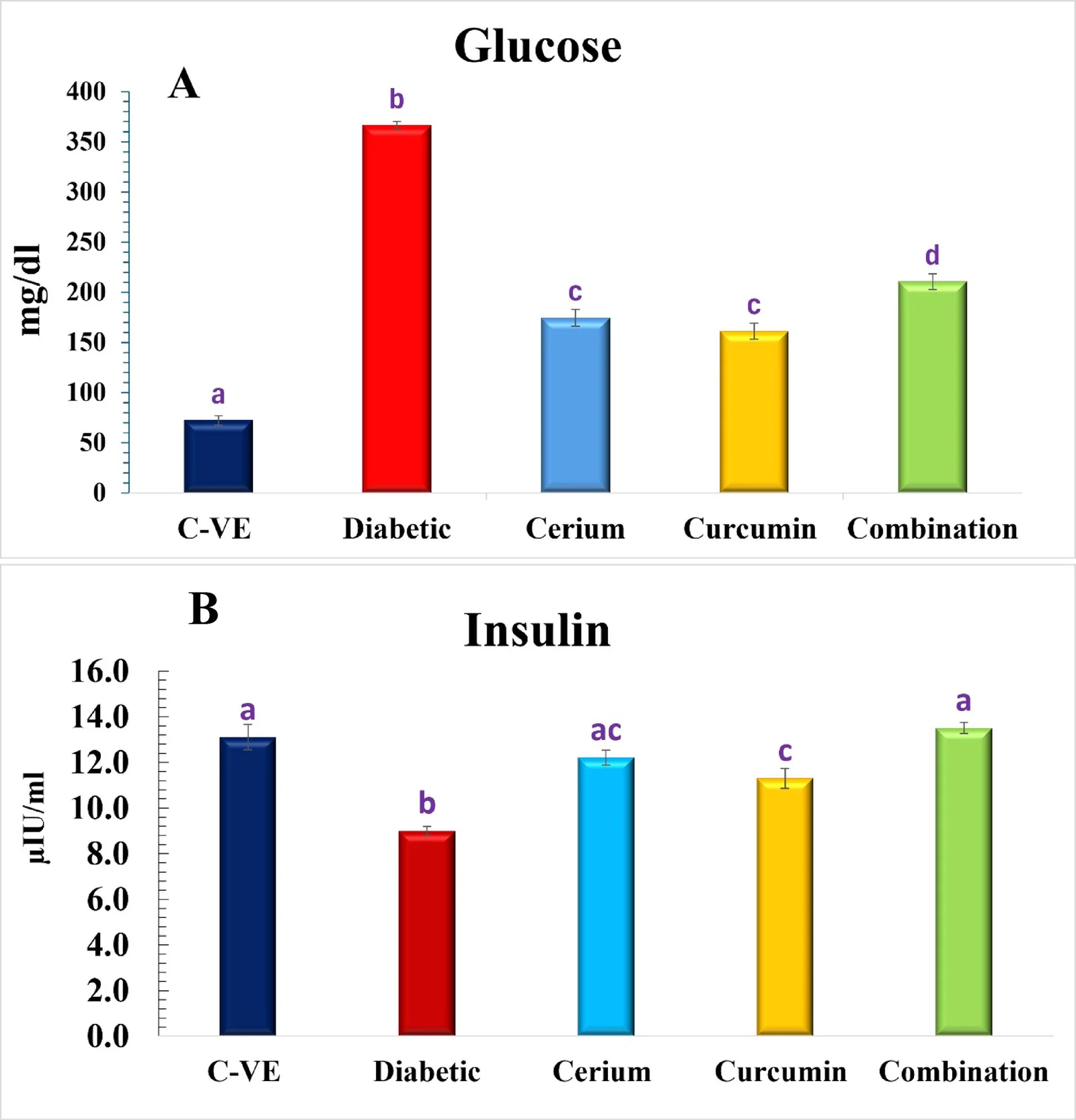
(**A**) Plasma glucose level. (**B**) Serum insulin level. All numerical findings are reported as the mean ± SE. To delineate statistical variations among cohorts, an alphabetical convention was adopted, where divergent letters denote a significant difference (*p* < 0.05) and identical letters signify statistical equivalence.

As illustrated in Fig. 4, the induction of diabetes resulted in a statistically significant elevation in plasma glucose levels and a concurrent reduction in serum insulin concentrations relative to the negative control group (*p* < 0.05). Monotherapy with CNPs significantly ameliorated this glycemic disruption, reducing blood glucose by approximately 55% and restoring insulin levels by 36% compared to the diabetic group. Nanocurcumin administration demonstrated decreasing plasma glucose by 76% and increasing insulin by 26%. Notably, the combinatorial administration of Nanocurcumin and CNPs induced a substantial glycemic correction, lowering glucose levels by 69% and elevating insulin by 50%. These findings highlight the antihyperglycemic potential of both agents, with the combination therapy exhibiting the major effect in functional insulin restoration.

### Effects of nanocurcumin and/or cerium oxide nanoparticles (CNPs) on lipid profile

**Fig. 5:**
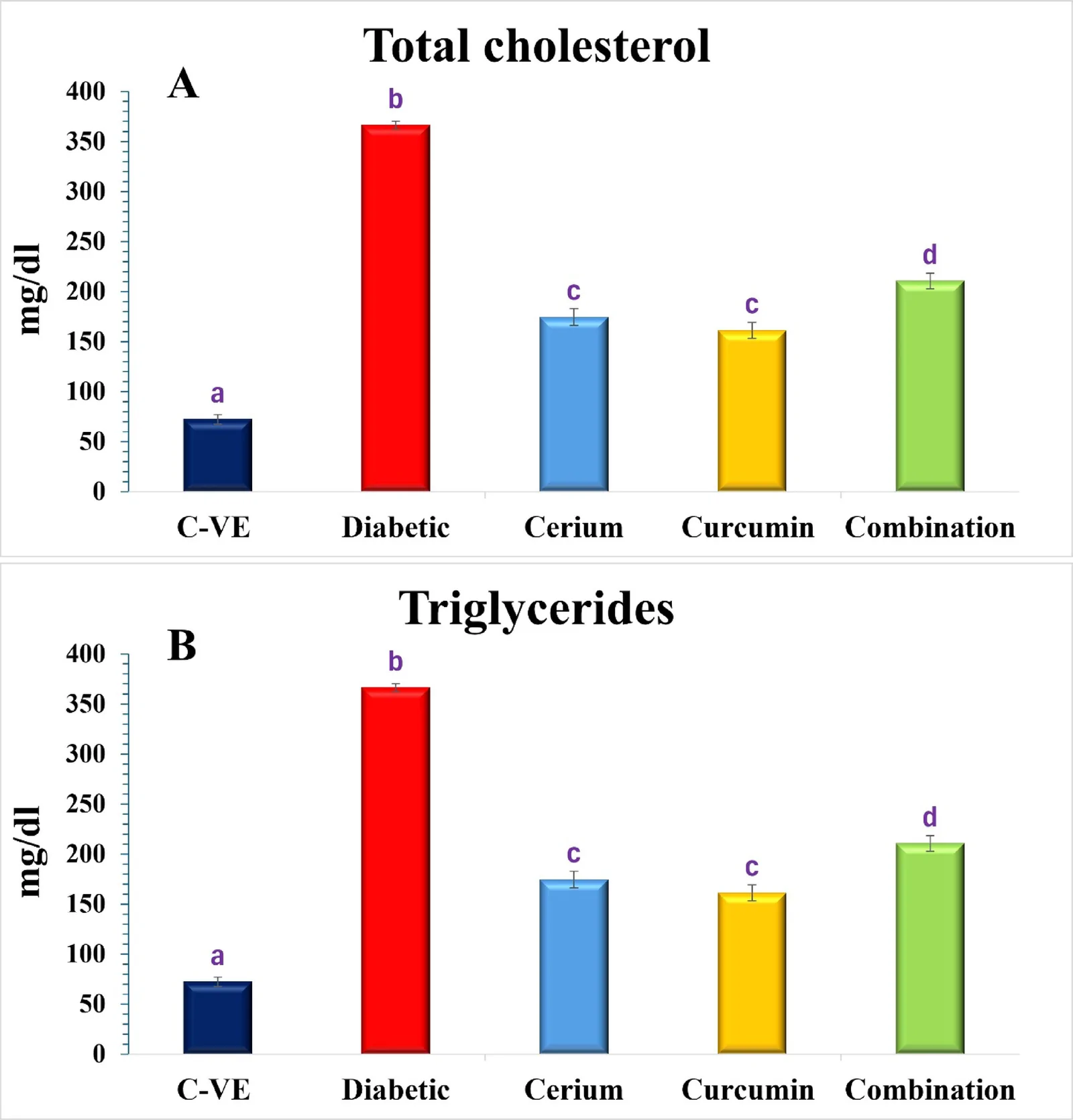
(**A**) Blood total cholesterol and (**B**) triglycerides in serum samples. All numerical findings are reported as the mean ± SE. To delineate statistical variations among cohorts, an alphabetical convention was adopted, where divergent letters denote a significant difference (*p* < 0.05) and identical letters signify statistical equivalence.

The experimental induction of diabetes elicited marked dyslipidemia, characterized by significant elevations in serum total cholesterol and triglycerides relative to the control group (Fig. 5). Therapeutic intervention with CNPs significantly improved lipid parameters, yielding reductions of 45% in total cholesterol and 52% in triglycerides compared to diabetic animals. Nanocurcumin exerted comparable hypolipidemic effects, reducing total cholesterol and triglycerides by approximately 50% and 56%, respectively. Furthermore, the combined administration significantly ameliorated dyslipidemia, lowering total cholesterol by 44% and triglycerides by 43%. The combinatorial approach demonstrated an enhanced capacity to regulate lipid metabolism, restoring these parameters to levels that were statistically comparable to the negative controls.

### Effects of nanocurcumin and/or cerium oxide nanoparticles (CNPs) on the liver function

**Fig. 6:**
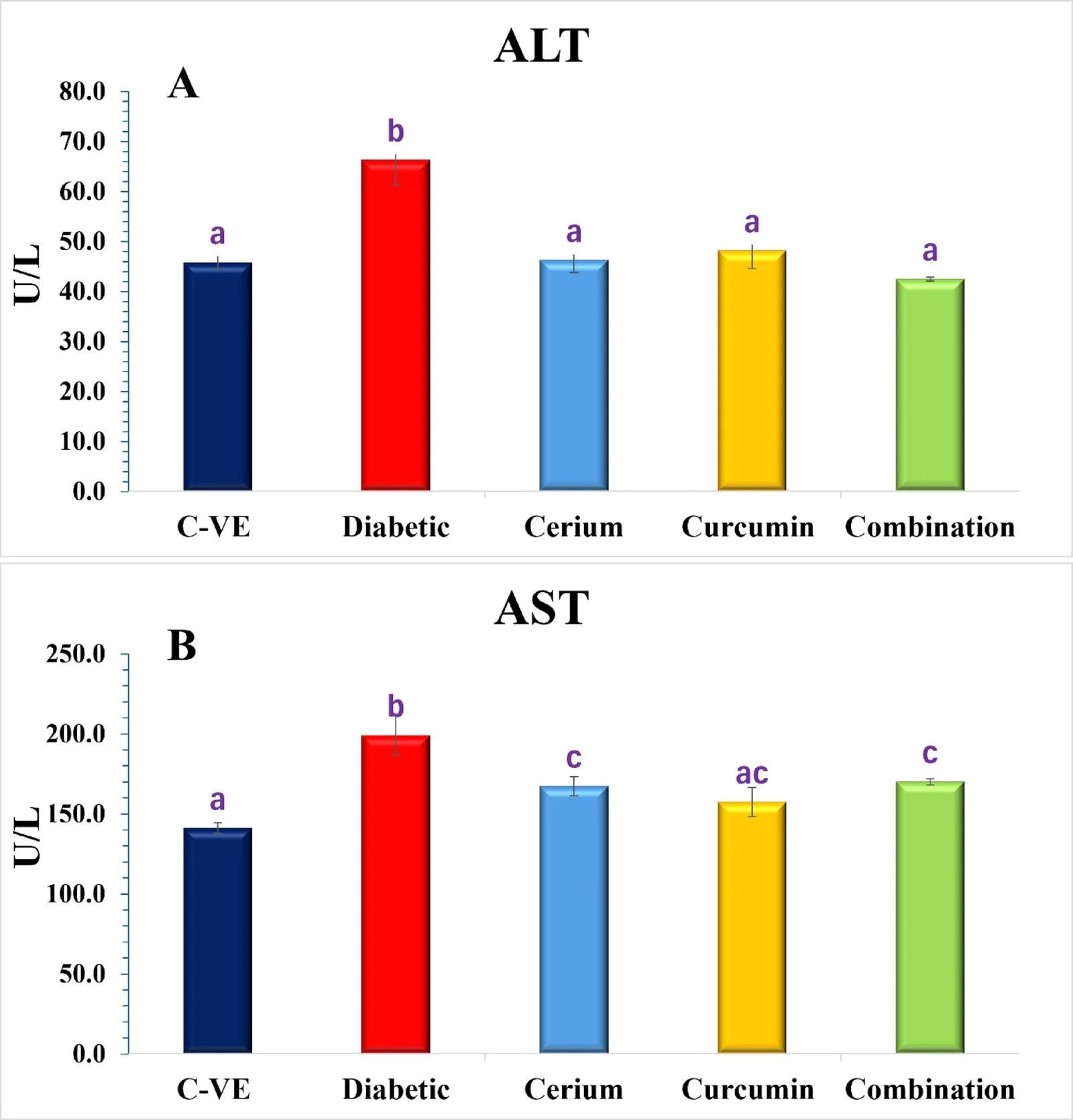
(**A**, **B**): Liver function tests in the serum of rats (**A**. ALT, **B**. AST). All numerical findings are reported as the mean ± SE. To delineate statistical variations among cohorts, an alphabetical convention was adopted, where divergent letters denote a significant difference (*p* < 0.05) and identical letters signify statistical equivalence.

Diabetes induction caused significant hepatic dysfunction, as reflected by marked elevations in serum ALT and AST levels when compared with the control group (Fig. 6). Treatment with CNPs significantly attenuated liver injury, reducing ALT and AST activities by approximately 30% and 16%, respectively, relative to diabetic animals. Nanocurcumin exerted comparable hepatoprotective effects, lowering ALT by 27% and AST by 21%. Notably, combined administration resulted in the greatest reduction in ALT (36%), restoring values to near-normal levels, while AST levels were reduced by about 15%. These findings demonstrate a protective effect on hepatic integrity.

### Effects of nanocurcumin and/or cerium oxide nanoparticles (CNPs) on the kidney function

**Fig. 7:**
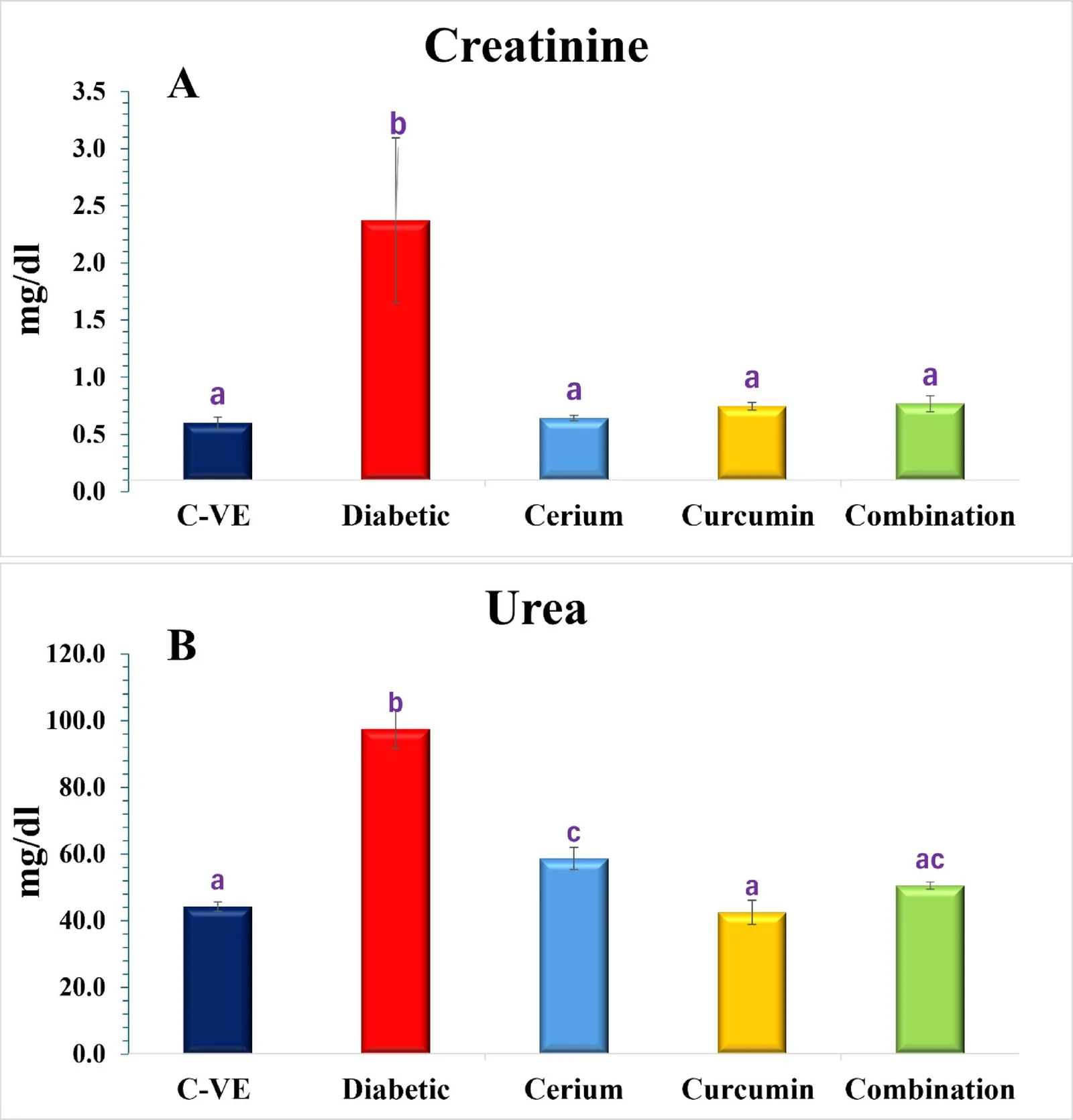
(**A**, **B**): Kidney function tests in the serum of rats (**A**. Creatinine, **B**. Urea). All numerical findings are reported as the mean ± SE. To delineate statistical variations among cohorts, an alphabetical convention was adopted, where divergent letters denote a significant difference (*p* < 0.05) and identical letters signify statistical equivalence.

The results presented in Fig. 7 indicate that diabetes markedly impaired renal function, as indicated by elevated serum urea and creatinine levels. Treatment with Cerium oxide nanoparticles, nanocurcumin, and their combination significantly improved kidney function, reducing urea by 40%, 56%, and 48%, respectively, and creatinine by 73%, 69%, and 68%, respectively, when compared with diabetic rats.

### Effects of nanocurcumin and/or cerium oxide nanoparticles (CNPs) on thyroid function

**Fig. 8:**
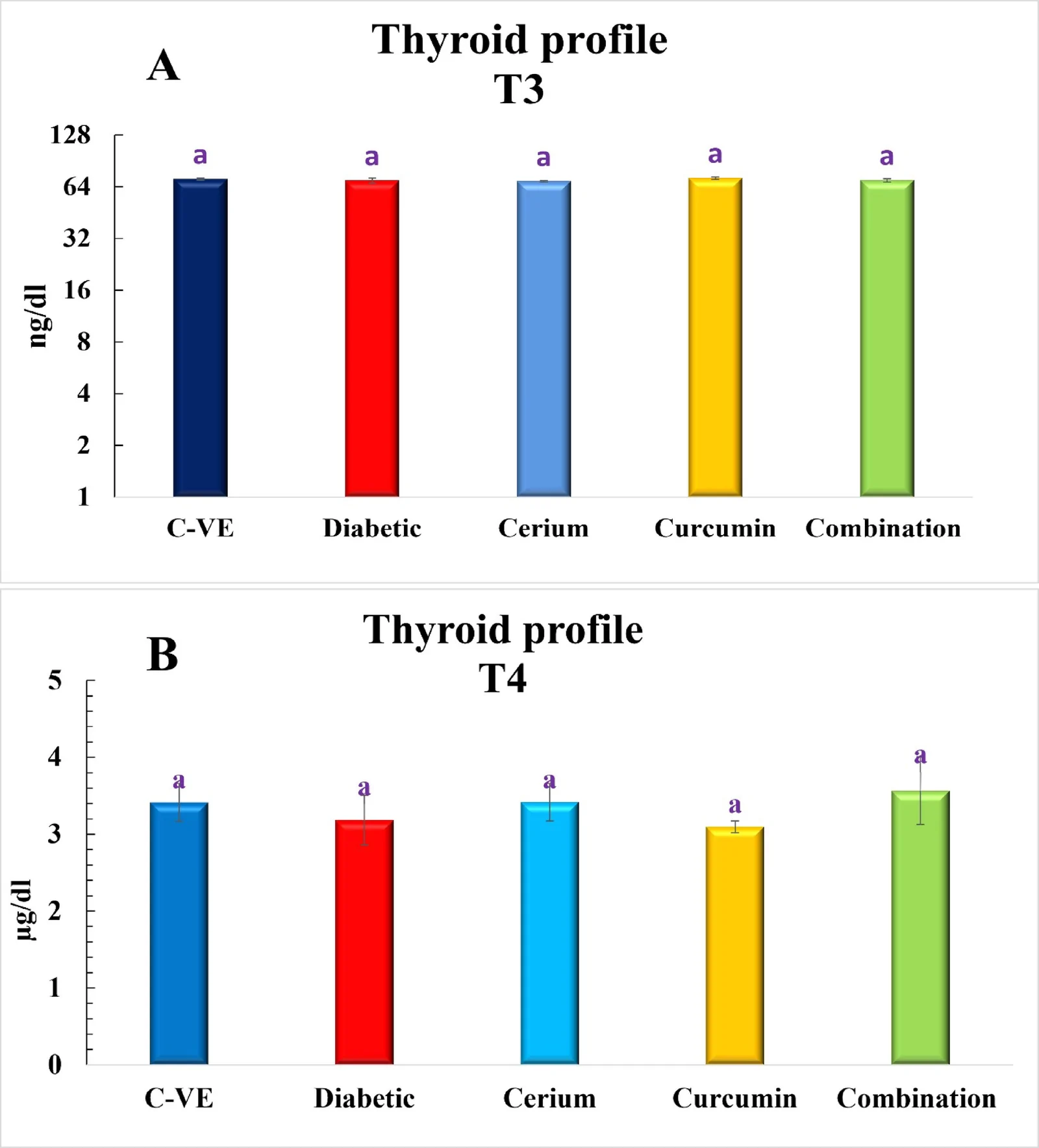
(**A**, **B**): Thyroid Function Profile (**A**. T3 and **B**. T4) across experimental groups. All numerical findings are reported as the mean ± SE. To delineate statistical variations among cohorts, an alphabetical convention was adopted, where divergent letters denote a significant difference (*p* < 0.05) and identical letters signify statistical equivalence.

Evaluation of thyroid function parameters, specifically T3 and T4, demonstrated that the induction of diabetes did not result in significant alterations in thyroid hormone homeostasis in this experimental model. As illustrated in Fig. 8. Serum levels of T3 and T4 in the diabetic group exhibited no significant statistical variance when contrasted with the negative control group (*p* > 0.05). Similarly, administration of Nanocurcumin did not induce any marked fluctuations in hormone levels, with values remaining consistent with both the control and diabetic groups. These findings suggest that the preservation of thyroid function was maintained across all experimental conditions studied.

### Effects of nanocurcumin and/or cerium oxide nanoparticles (CNPs) on complete blood picture (CBC)

**Fig. 9:**
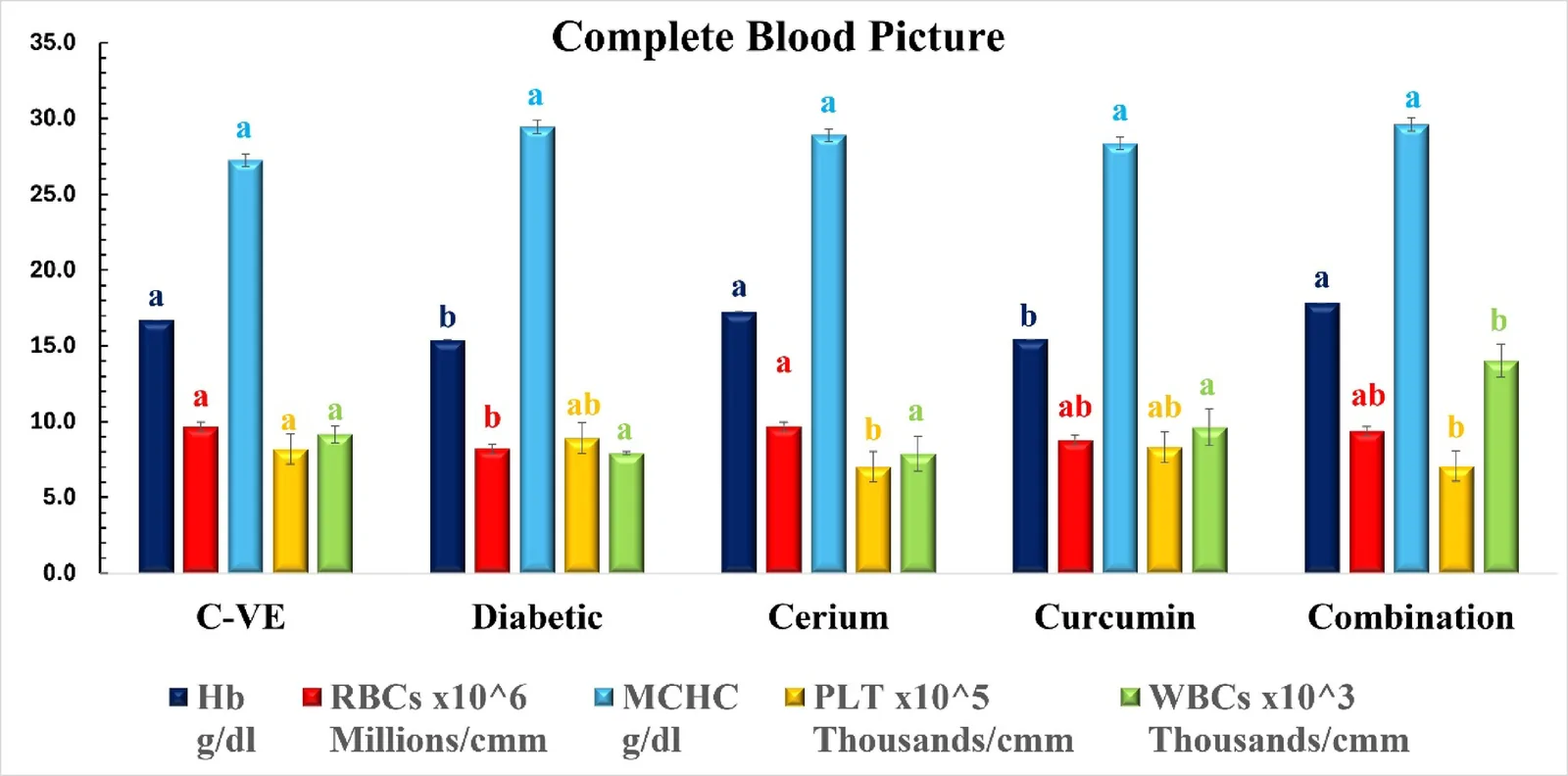
Complete Blood Count (CBC) Parameters of rats. All numerical findings are reported as the mean ± SE. To delineate statistical variations among cohorts, an alphabetical convention was adopted, where divergent letters denote a significant difference (*p* < 0.05) and identical letters signify statistical equivalence.

The evaluation of the CBC revealed specific modulations across the experimental groups, with a primary focus on the therapeutic deviations from the diabetic baseline (Fig. 9). The induction of diabetes caused a significant decline in Hb concentrations compared to the negative control group (b vs. a). Both CNPs and the combination therapy successfully restored Hb levels to match the control group (‘a’), whereas Nanocurcumin did not induce a statistically significant difference when compared to the diabetic baseline (‘b’). Analysis of RBC counts also demonstrated a significant therapeutic response. While the diabetic group exhibited a significantly lower baseline (‘b’), CNPs effectively elevated RBC counts to normal levels (‘a’). Both Nanocurcumin and the Combination therapy showed an intermediate RBC profile (‘ab’), indicating no significant statistical deviation from either the negative control or the diabetic state. The MCHC remained stable across all groups, with no significant differences observed (*p* > 0.05). Additionally, Platelet counts varied; while the diabetic and Nanocurcumin groups exhibited overlapping significance with the negative control (‘ab’), both the Cerium and Combination treatments showed significant differences in contrast to the negative control group (‘b’ vs. ‘a’). Regarding WBCs, the combination treatment resulted in a marked elevation in counts in contrast to all other evaluated groups (*p* < 0.05), indicating a heightened leukocytic mobilization specific to the concurrent administration. In contrast, the diabetic, Nanocurcumin, and CNPs groups showed no significant variation in WBC levels relative to the control (all sharing the letter ‘a’ (Fig. 9).

### Effects of nanocurcumin and/or cerium oxide nanoparticles (CNPs) on oxidative/antioxidative status markers of hepatic tissues

**Fig. 10:**
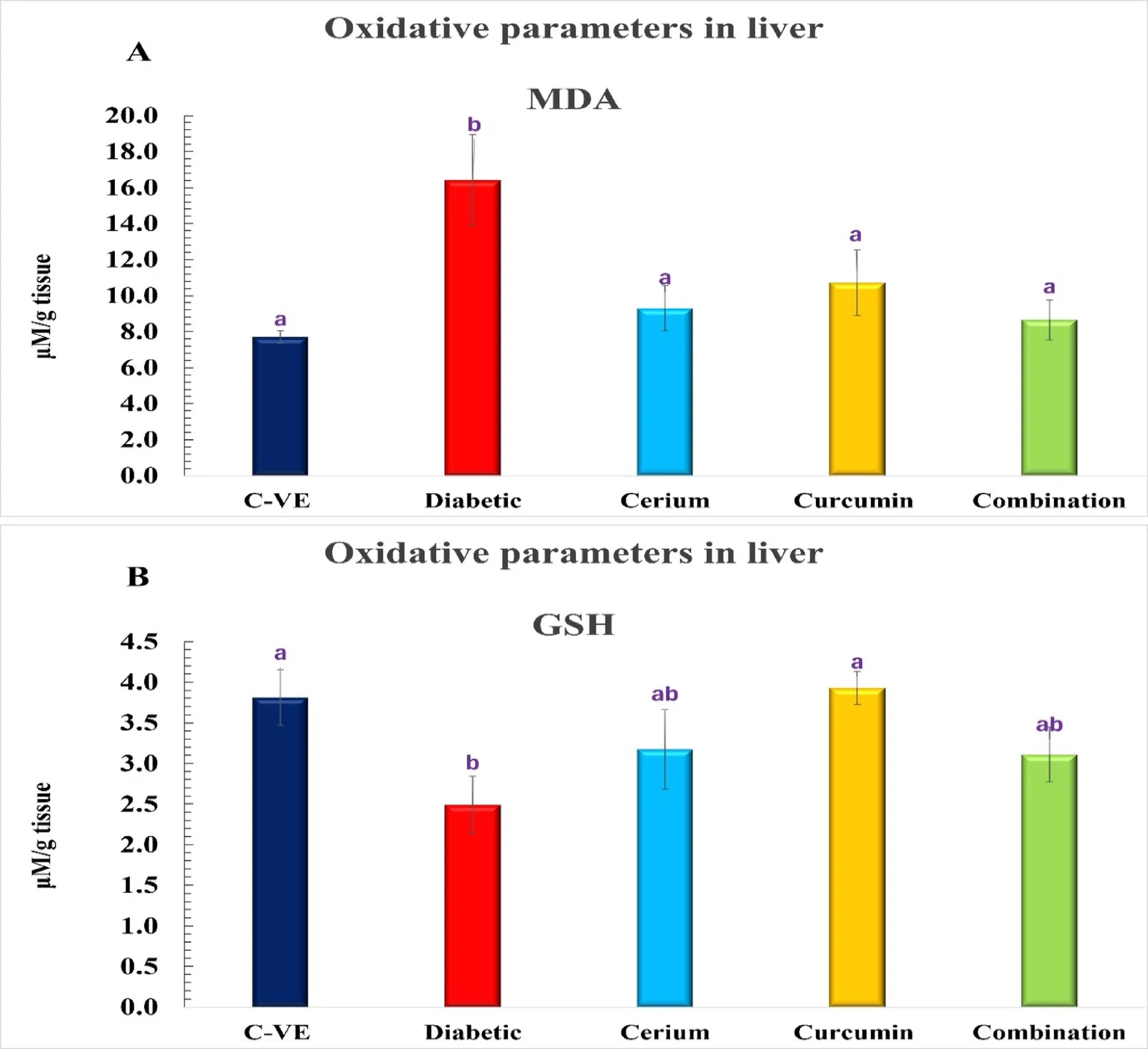

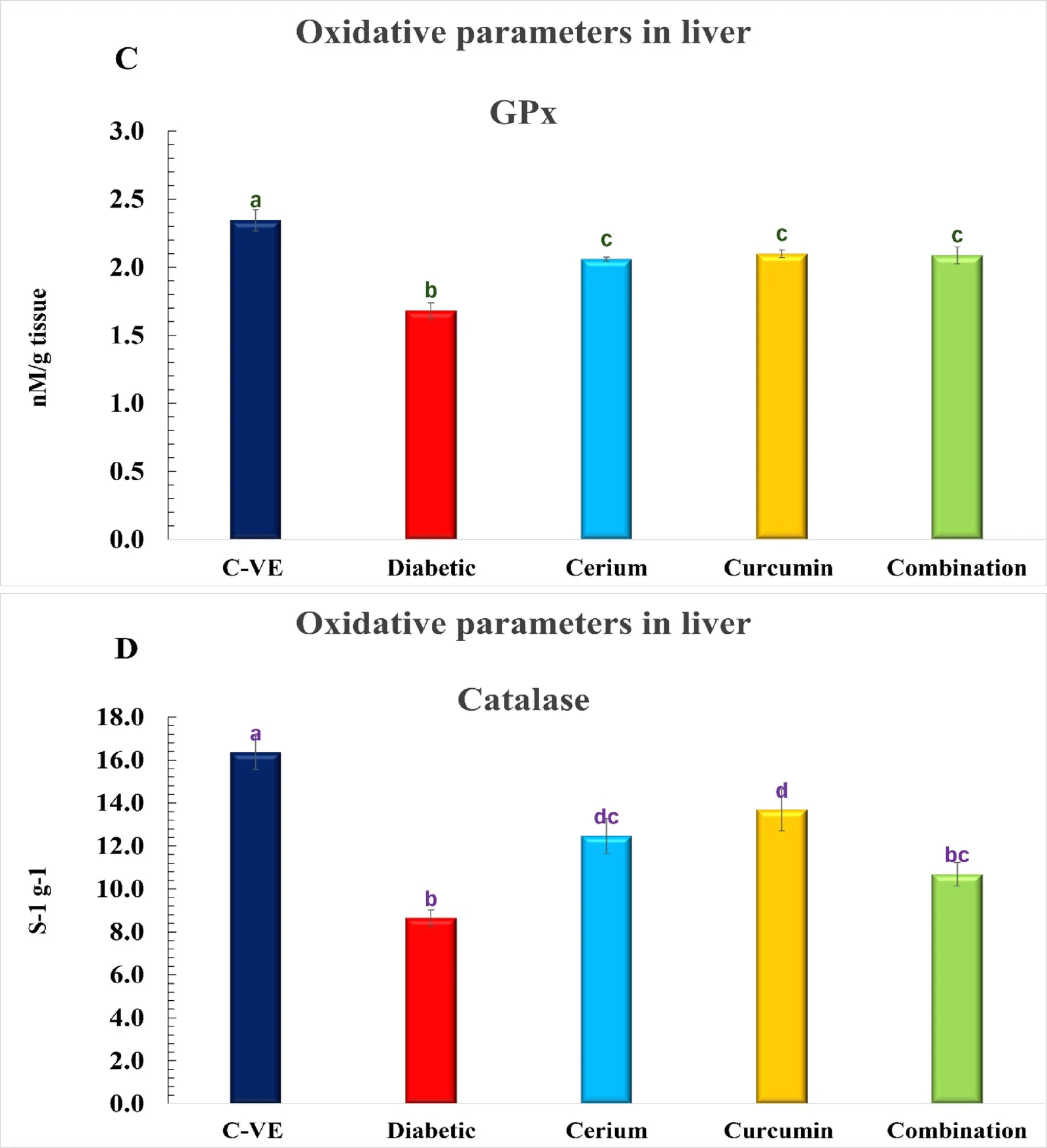
(**A**, **B**, **C**, **D**): Levels of MDA and antioxidant parameters (**A**. MDA, **B**. GSH, **C**. GPx, **D**. Catalase) in the liver tissue of rats. All numerical findings are reported as the mean ± SE. To delineate statistical variations among cohorts, an alphabetical convention was adopted, where divergent letters denote a significant difference (*p* < 0.05) and identical letters signify statistical equivalence.

Diabetes significantly exacerbated oxidative stress in hepatic tissues, as evidenced by elevated MDA concentrations and concurrent reductions in GSH levels, as well as GPx and catalase activities (Fig. 10). Interventions with CNPs, nanocurcumin, and their combination significantly attenuated lipid peroxidation, reducing hepatic MDA levels by 35–47% relative to diabetic rats. This attenuation was accompanied by a restoration of antioxidant defenses, demonstrated by increases in GSH (25–58%), GPx activity (23–25%), and catalase activity (24–58%) across the treated groups. Overall, the nano-based treatments effectively mitigated diabetes-induced hepatic oxidative stress.

### Effects of nanocurcumin and/or cerium oxide nanoparticles (CNPs) on oxidative/antioxidative status markers of pancreatic tissues

**Fig. 11:**
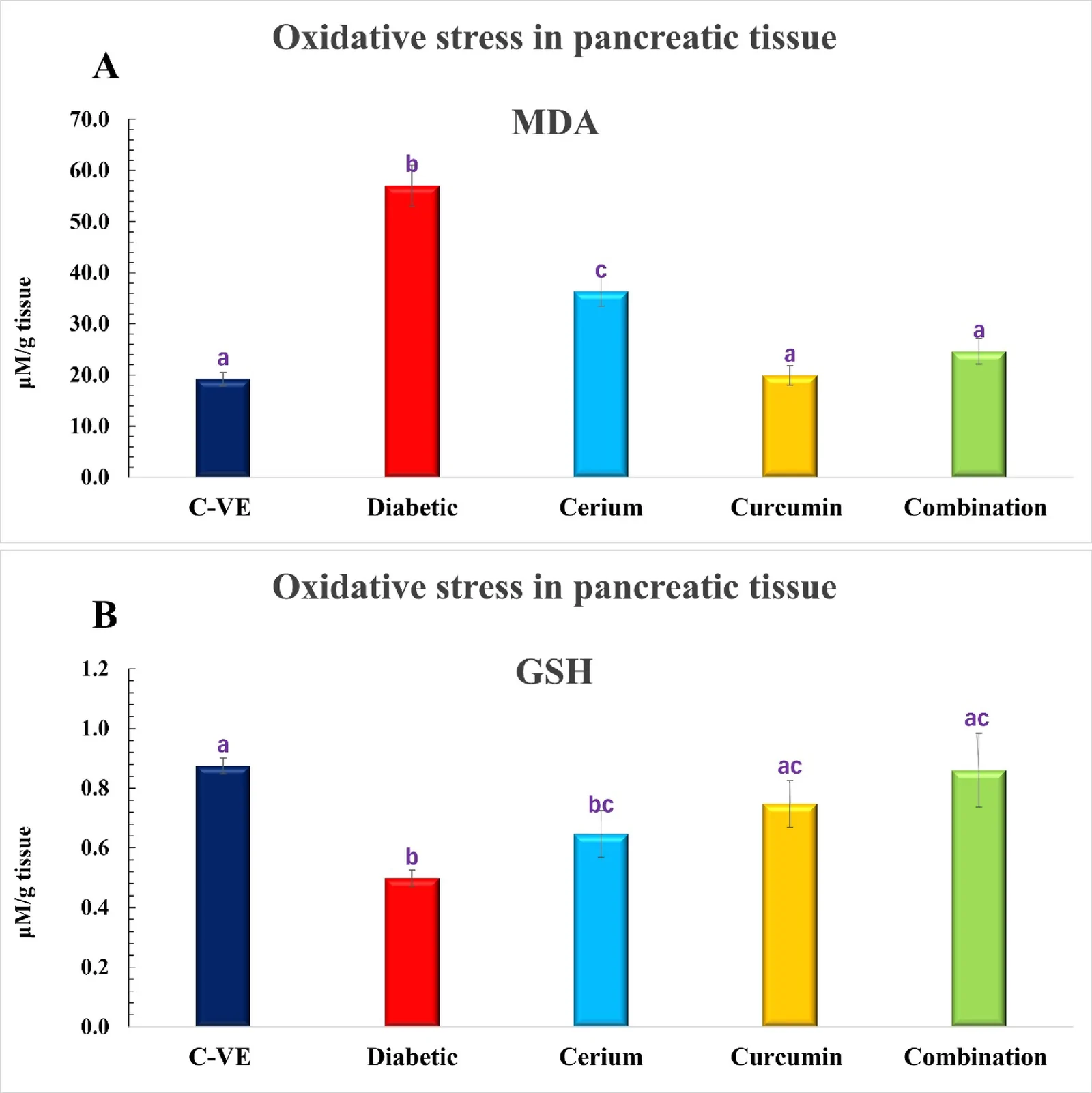

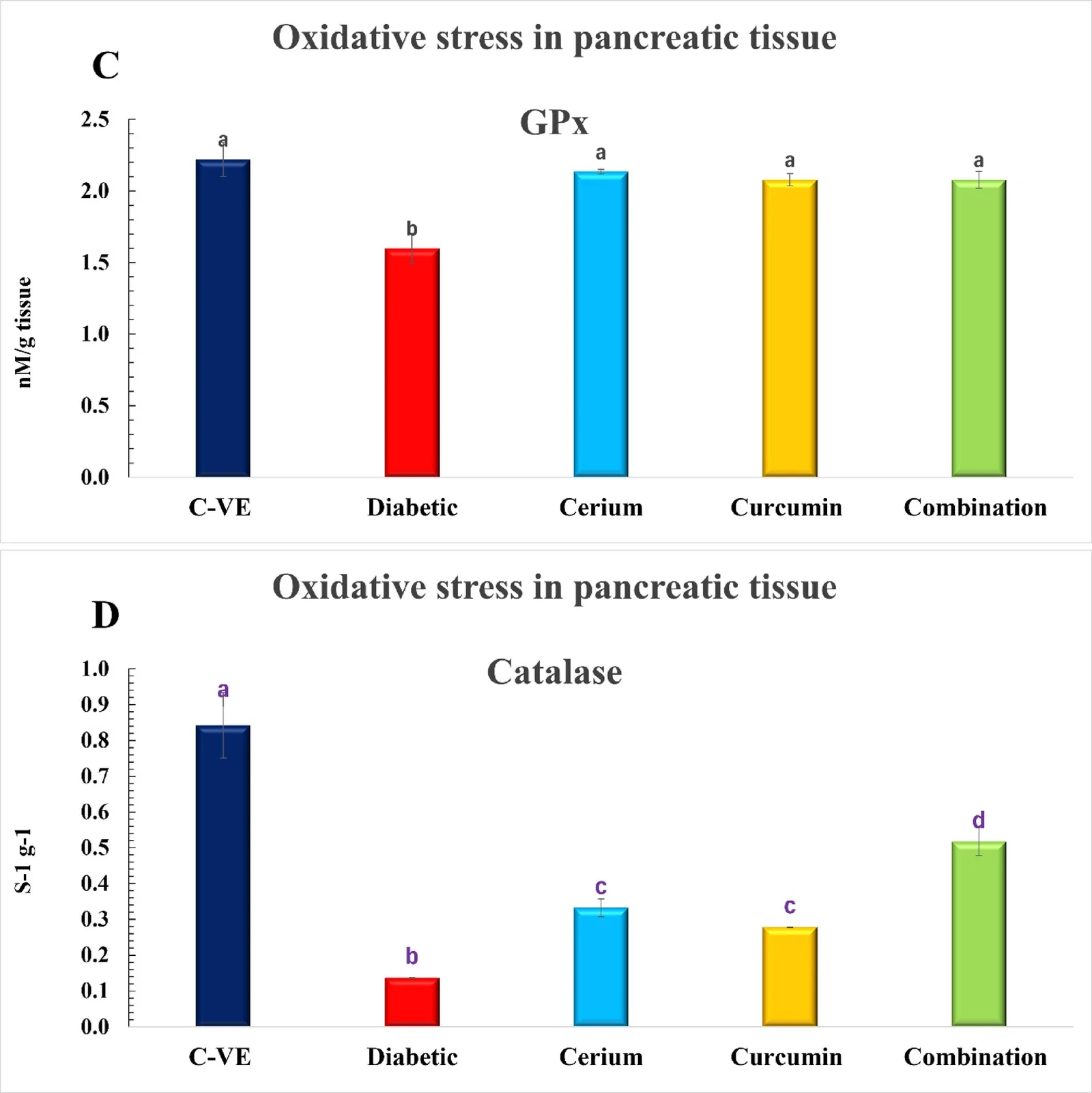
(**A**, **B**, **C**, **D**) Levels of MDA and antioxidant parameters (**A**. MDA, **B**. GSH, **C**. GPx, and **D**. Catalase) in the pancreatic tissue of rats. All numerical findings are reported as the mean ± SE. To delineate statistical variations among cohorts, an alphabetical convention was adopted, where divergent letters denote a significant difference (*p* < 0.05) and identical letters signify statistical equivalence.

In pancreatic tissues, the diabetic state induced a marked increase in oxidative stress, demonstrated by a significant accumulation of MDA and a depletion of GSH, GPx, and catalase activities (Fig. 11). Nano-therapeutic treatments significantly suppressed localized lipid peroxidation, reducing pancreatic MDA levels by 36–65% compared to the diabetic group. This reduction was coupled with a substantial restoration of endogenous antioxidant defenses, including increased GSH, GPx, and catalase activities. Curcumin and combination groups were better in MDA reduction and GSH elevation. While the combination group was the best in the elevation of catalase activity.

### Effects of nanocurcumin and/or cerium oxide nanoparticles (CNPs) on hepatic insulin signaling gene expression

**Fig. 12:**
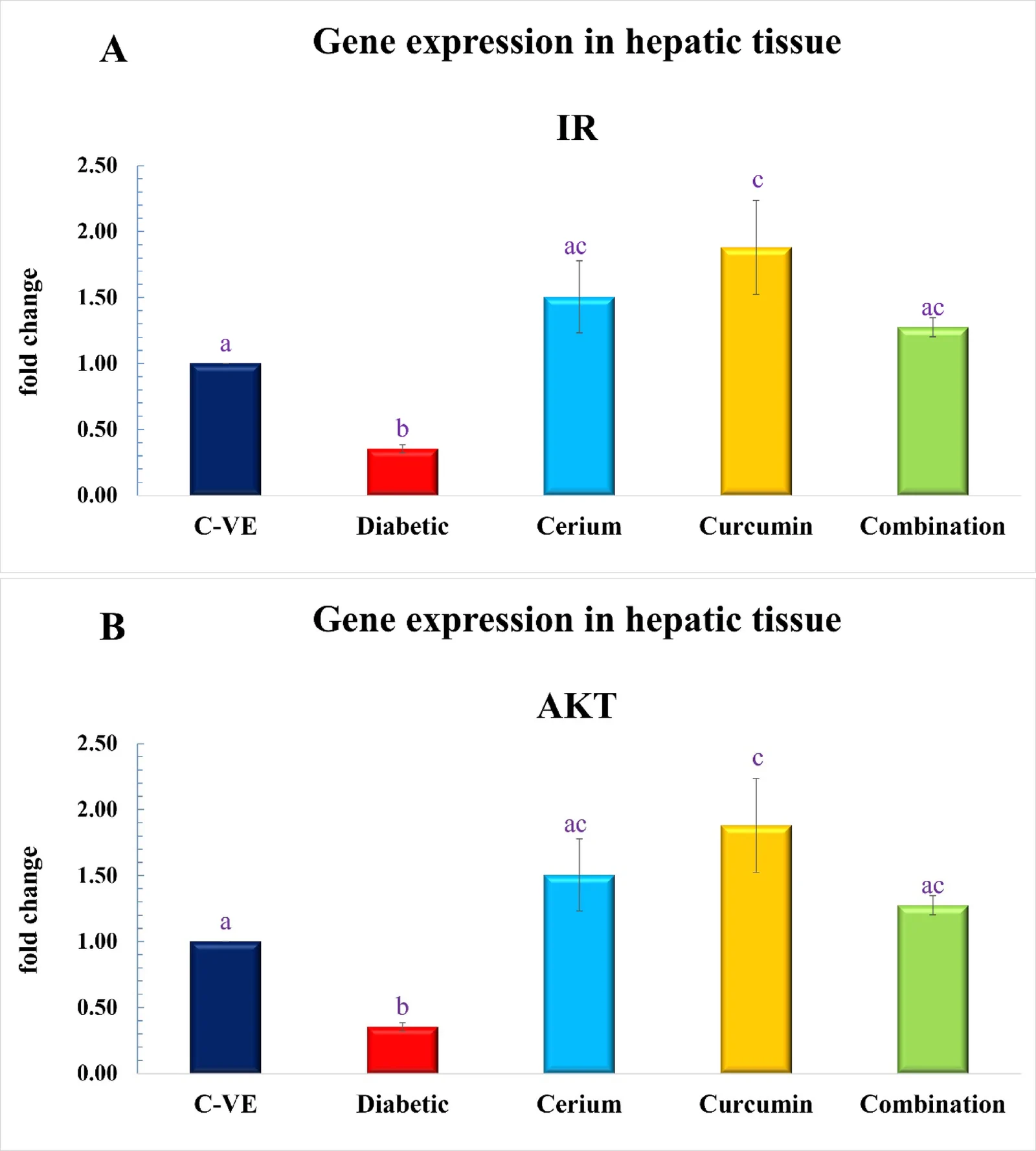

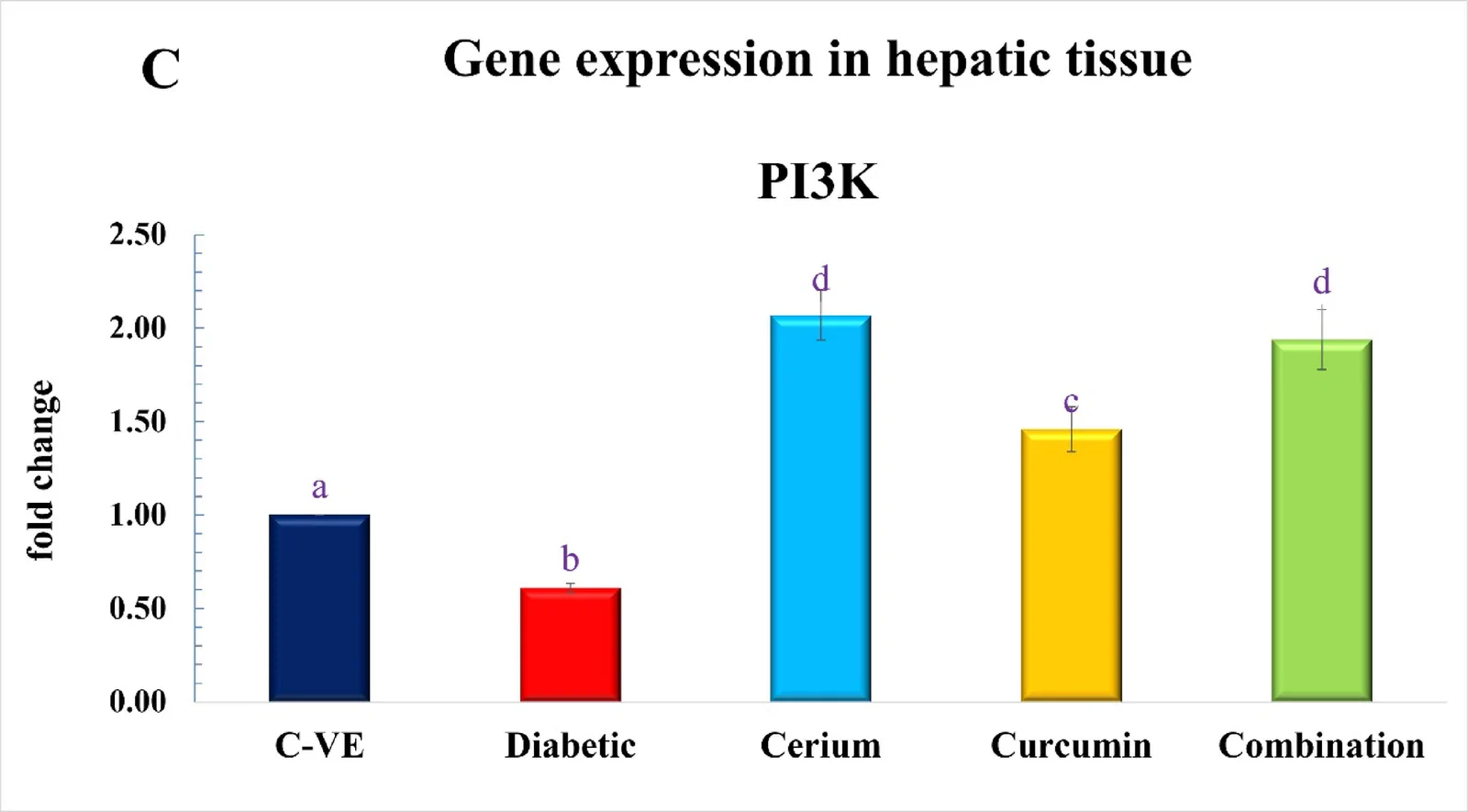
Levels of gene expression in liver tissue (**A**. IR, **B**. AKT, **C**. PI3K). All numerical findings are reported as the mean ± SE. To delineate statistical variations among cohorts, an alphabetical convention was adopted, where divergent letters denote a significant difference (*p* < 0.05) and identical letters signify statistical equivalence.

Molecular analyses indicated that diabetes markedly suppressed the hepatic insulin signaling cascade, evidenced by reduced mRNA expression of IR, AKT, and PI3K relative to controls (Fig. 12). All treatments significantly upregulated the mRNA expression of IR, AKT, and PI3K compared to the diabetic group. Treatment with CNPs significantly upregulated pathway activation, increasing AKT and PI3K expression by approximately 4.3-fold and 3.4-fold, respectively, compared to diabetic animals. Nanocurcumin administration induced the most prominent activation of AKT, elevating its expression over five-fold, alongside a 2.4-fold increase in PI3K expression. Notably, the combined treatment restored the insulin signaling pathway, significantly upregulating IR and PI3K expression while simultaneously maintaining high AKT activation. These molecular findings confirm the effective reactivation of hepatic insulin-responsive pathways.

### Effects of nanocurcumin and/or cerium oxide nanoparticles (CNPs) on iNOS and VEGF gene expression in pancreatic tissue

**Fig. 13:**
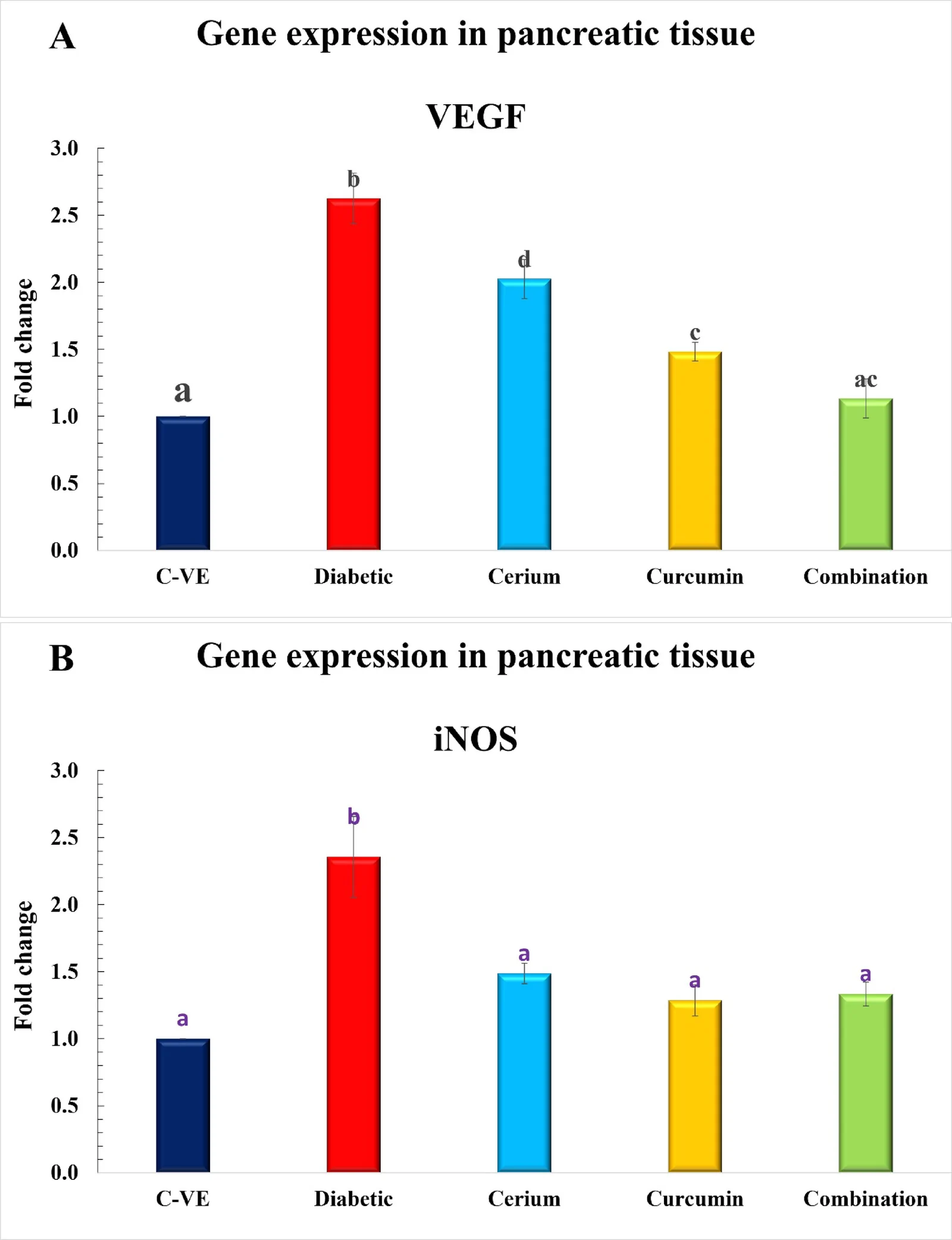
(**A**, **B**) The relative gene expression of (**A**) VEGF and (**B**) iNOS in pancreatic tissue. All numerical findings are reported as the mean ± SE. To delineate statistical variations among cohorts, an alphabetical convention was adopted, where divergent letters denote a significant difference (*p* < 0.05) and identical letters signify statistical equivalence.

Induction of diabetes markedly provoked pancreatic inflammation and vascular alteration. This condition was characterized by a marked increase in the expression of iNOS and VEGF genes by 2.36-fold and 2.63-fold, respectively, relative to the negative control group (set at 1.00). Treatment with CNPs partially alleviated these elevations, reducing iNOS and VEGF expression to 1.49- and 2.03-fold, respectively. Nanocurcumin administration exhibited superior efficacy when evaluated against CNPs, further suppressing iNOS levels to 1.29-fold and VEGF to 1.48-fold. Notably, the combined treatment reduced VEGF expression to 1.13-fold, nearly returning to control levels, and maintained a suppression of iNOS at 1.33-fold. These results indicate that while both agents are effective individually, the combination therapy offers the most comprehensive normalization of pancreatic gene expression profiles (Fig. 13).

### Histopathological examination

#### Light-microscopy findings

**Fig. 14:**
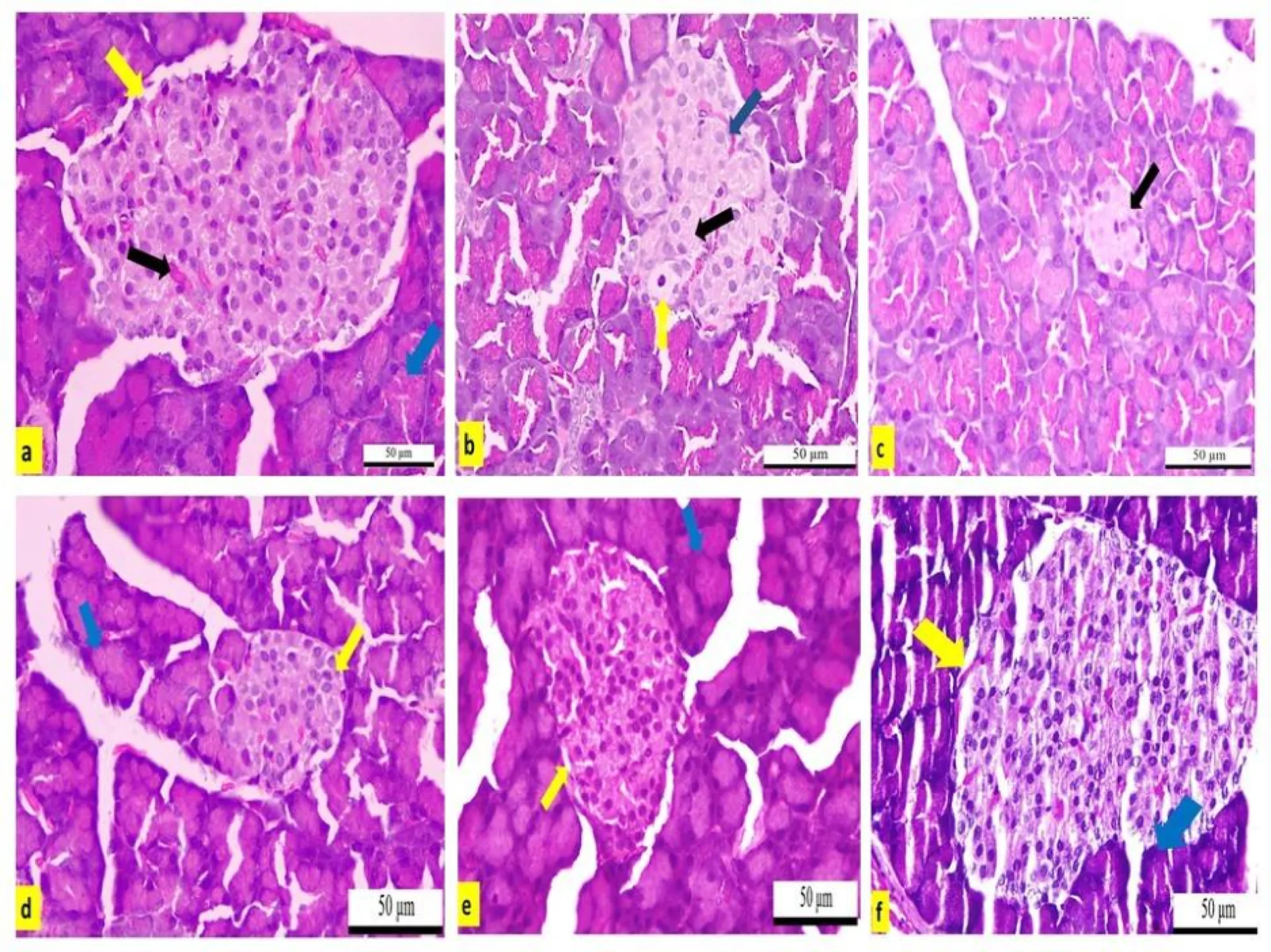
(**a**–**f**) Pancreatic sections from adult male albino rats. H&E.X400. (**a**) **The control group** exhibited unremarkable histological features, characterized by preserved architecture of both the exocrine acini (blue arrow) and the islets of Langerhans (yellow arrow). Notably, capillaries penetrating the islets demonstrated direct juxtaposition with the endocrine cell population (black arrow). (**b** & **c**) **The diabetic group** showing: (**b**) Histological examination of the pancreatic islets revealed three distinct pathological features: vacuolated and ballooned degenerative cells (yellow arrow), dilated and congested capillary networks (blue arrow), and homogenous acidophilic mass deposition within islet cells (black arrow). (**c**) Shrunken and atrophied islet. (**d**, **e**, **f**) **Curcumin & CNPs and the combination groups ** demonstrated an improvement in the islet structure (yellow arrow) and a relatively healthy exocrine acini structure, like the negative control group (blue arrow).

Microscopic observation of hematoxylin and eosin-stained pancreas sections from the control animals revealed typical structural integrity, with both the exocrine acini and the islets of Langerhans appearing morphologically intact. The islets are identified from the exocrine tissue due to their paler appearance. They appeared as an irregular mass of cells with a delicate connective tissue boundary. The capillaries invading the islets are in direct contact with the endocrine cells of the islets (Fig. 14a) and (Table 2). Conversely, specimens from the diabetic cohort exhibited a range of pathological modifications within the islets of Langerhans when evaluated against the healthy controls, as follows: the islets had vacuolated and ballooned cells, and the capillaries exhibited marked dilatation and congestion. Moreover, deposition of a homogeneous acidophilic substance suggestive of amyloid in the cells of the islets; however, confirmatory staining was not performed (Fig. 14b) and (Table 2). Also, there was a shrunken and atrophied islet as an indication of degeneration (Fig. 14c) and (Table 2).

**Table 2:** Pathological scoring of the diabetes lesions on the pancreas in different treated groups based on the degree of vacuolation and acidophilic mass deposition in the cells of the islet, dilated and congested capillaries, and atrophied islets.

Groups	Vacuolation and acidophilic mass deposition in the cells of the islet (Scores 0–4)	Dilated and congested capillaries (Scores 0–3)	Atrophied islets (Scores 0–3)
Control	0.0 ± 0^**b**^	0.0 ± 0^**b**^	0.0 ± 0^**b**^
Diabetic	2.8 ± 0.3^**a**^	2.5 ± 0.3^**a**^	2.7 ± 0.3^**a**^
Curcumin	0.5 ± 0.2^**b**^	0.3 ± 0.3^**b**^	0.3 ± 0.3^**b**^
Cerium	0.3 ± 0.2^**b**^	0.3 ± 0.3^**b**^	0.3 ± 0.3^**b**^
Combination	0.0 ± 0^**b**^	0.0 ± 0^**b**^	0.0 ± 0^**b**^

The data were statistically analyzed by one-way ANOVA, followed by the LSD post hoc test. Data were presented as mean ± SEM. Means with different superscripts within the same column were significantly different at *p* < 0.0001.

On the other hand, the treated groups with curcumin or CNPs or the combination between them demonstrated a valuable improvement in the cells of the islets of Langerhans with decreased necrosis and reduction. Moreover, reduced dilatation and congestion of the capillaries with minimization of acidophilic mass accumulation within the cells of the islets and a relatively healthy exocrine acini structure, like the negative control group (Fig. 14d, e, f) and (Table 2), respectively.

**Fig. 15:**
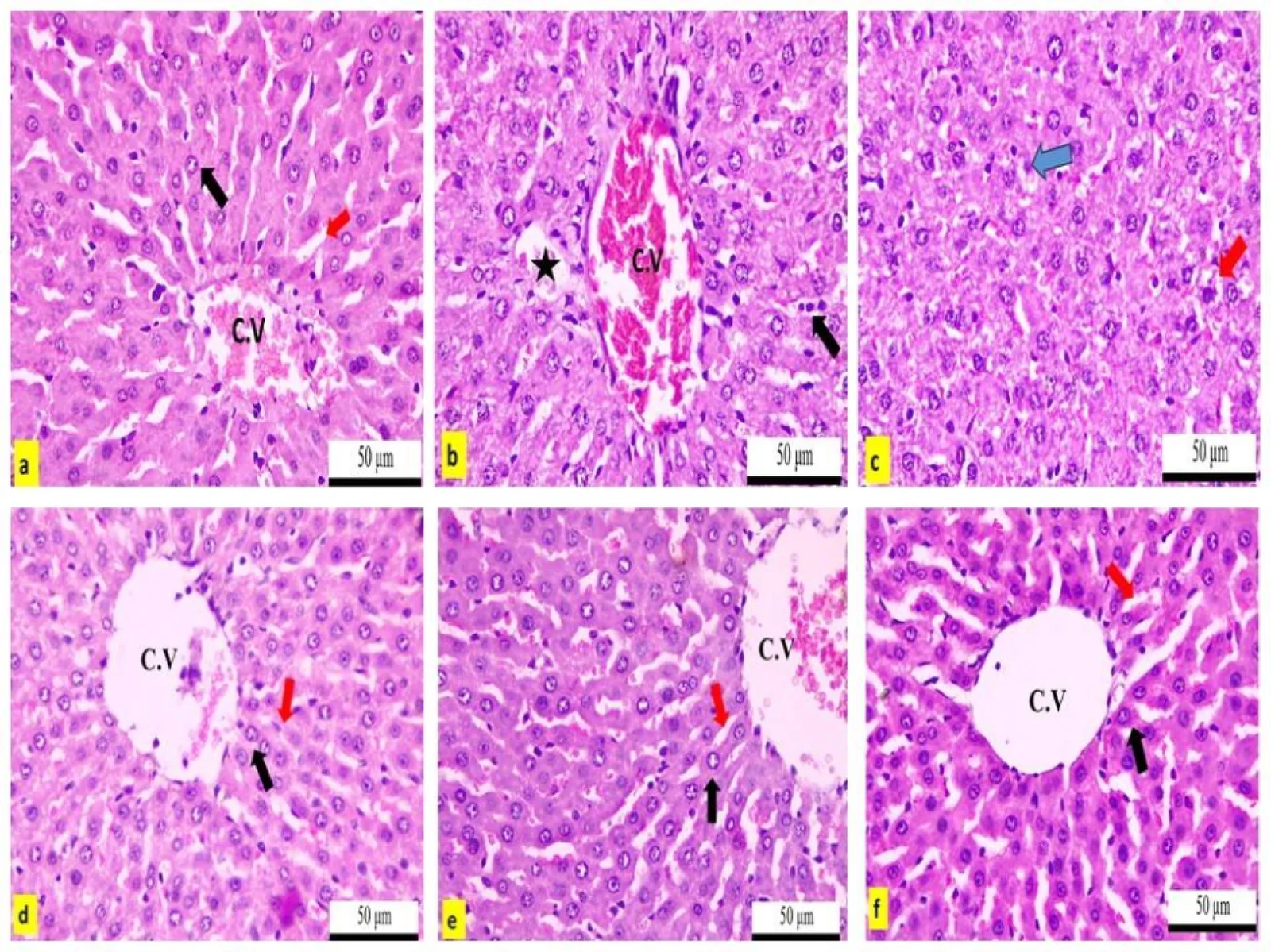
(**a**–**f**) Liver sections from adult male albino rats. H & E.X400. (**a**) Histological examination of the control group demonstrated the characteristic hepatic architecture, featuring anastomosing cords of normal polyhedral hepatocytes (black arrow) radiating outward from an unremarkable central vein (CV). These cellular plates were separated by patent hepatic sinusoids exhibiting typical morphology (red arrow). (**b**, **c**) The diabetic group: (**b**) The pathological specimen exhibited marked vascular alterations, including severe congestion and dilatation of both the central vein (CV) and hepatic sinusoids (black star). This was accompanied by disruption of the normal hepatocyte cord architecture and the presence of numerous Kupffer cells (black arrow). Furthermore, distinct hepatocellular changes were observed: some cells demonstrated ballooning degeneration with cytoplasmic vacuolization (red arrow), while others showed overt cellular degeneration characterized by karyolysis (blue arrow). (**c**) Also, some hepatocytes display ballooning and cytoplasmic vacuolization (red arrow), while other hepatocytes displayed degeneration and karyolysis (blue arrow). (d&e&f) Curcumin & cerium and the combination groups exhibited normal architecture of the central vein (C.V) and hepatocytes (black arrow) separated by normal hepatic sinusoids (blue arrow).

Histological assessment of the control group's H&E-stained liver specimens demonstrated typical parenchymal integrity, characterized by a standard hepatic microarchitecture, which is formed of cords of polyhedral hepatocytes, with the histological architecture distinguished by a centrally positioned vesicular nucleus radiating outward from a typical central vein, with intervening healthy blood sinusoids providing clear demarcation between these structures (Fig. 15a and Table 3). The diabetic group exhibited severe damage that was observed as congestion and dilatation of the central vein and blood sinusoids. Also, disorganization in the hepatic cords with numerous Kupffer cells. In addition, some hepatocytes exhibited ballooning and vacuolization while others displayed karyolysis and degeneration (Fig. 15b, c) and (Table 3). Interestingly, the hepatic tissue of the treated groups with curcumin, cerium, or the combination of both showed normal organization of the hepatocytes and less congested central veins and blood sinusoids. Hepatocytes were nearly normal in comparison to the diabetic group (Fig. 15d, e, f) and (Table 3).

**Table 3:** Pathological scoring of the diabetes lesions on the liver tissue of different treated groups based on the degree of congestion and dilatation of the central vein and the sinusoids, disorganization of the hepatic architecture, hepatocyte ballooning, and degeneration.

Groups	Congestion and dilation of C.V(Scores 0–3)	Disorganization of hepatic cords (Scores 0–3)	Vacuolization and ballooning of hepatocytes (Scores 0–3)
Control	0.0 ± 0^b^	0.0 ± 0^b^	0.0 ± 0^b^
Diabetic	2.8 ± 0.3^a^	2.8 ± 0.3^a^	2.3 ± 0.5^a^
Curcumin	0.3 ± 0.3^b^	0.5 ± 0.3^b^	0.5 ± 0.3^b^
Cerium	0.5 ± 0.3^b^	0.3 ± 0.3^b^	0.3 ± 0.3^b^
Combination	0.0 ± 0^b^	0.0 ± 0^b^	0.0 ± 0^b^

The data were statistically analyzed by one-way ANOVA, followed by the LSD post hoc test. Data were presented as mean ± SEM. Means with different superscripts within the same column were significantly different at *p* < 0.0001.

#### Immunohistochemical findings

**Fig. 16:**
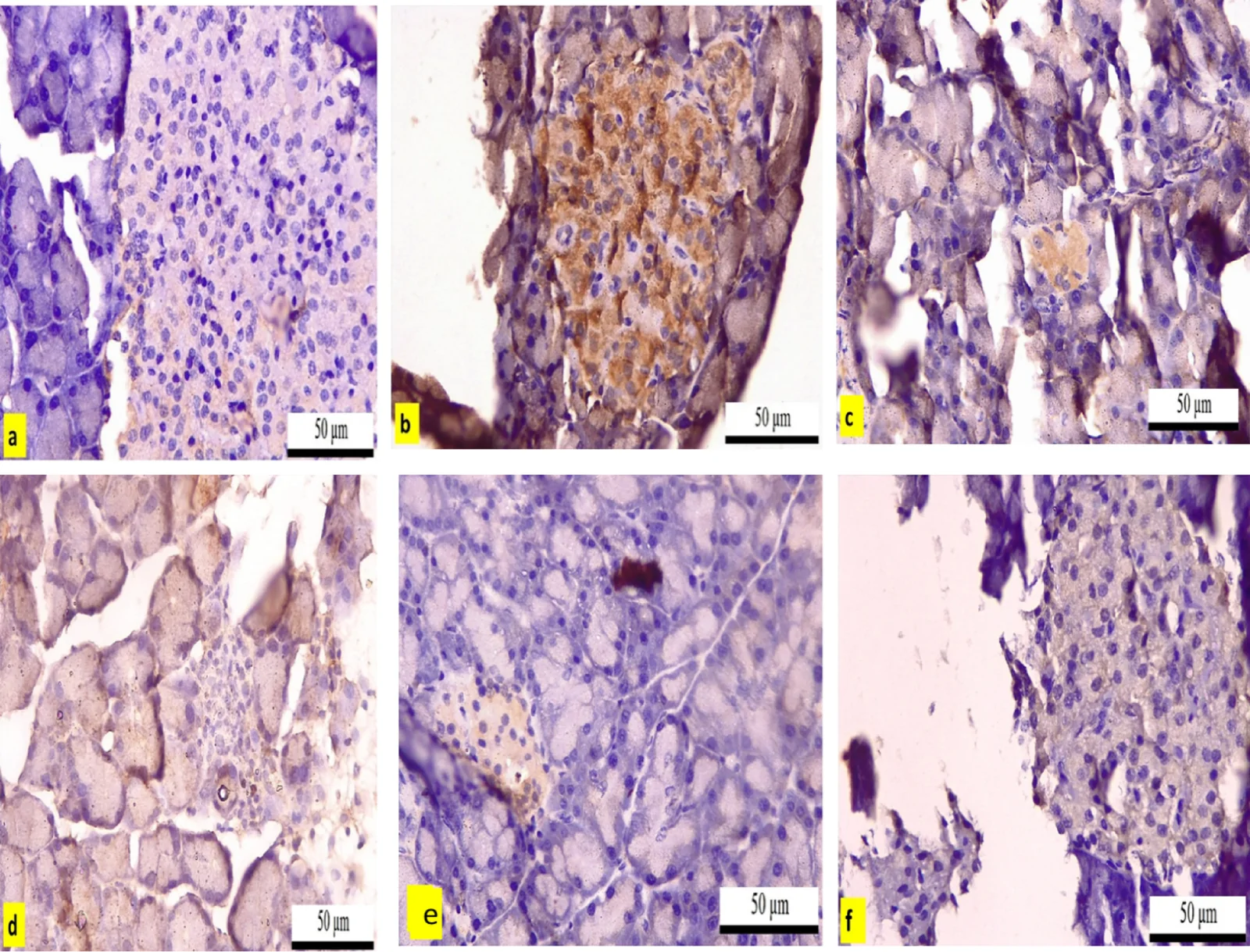
Demonstrating immune expression of NF-κBp65 stained pancreatic sections (× 400). (**a**–**f**) (**a**) The control group exhibited a negligible NF-κBp65 immunoreaction. (**b**, **c**) the diabetic group revealed: (**b**) intense NF-κBp65 staining in most cells of the islets and the pancreatic tissue (**c**) atrophied islets showing positive reaction. (**d,** **e**, **f**) curcumin & cerium, and the combination groups showed a weak expression of the NF-κBp65, respectively.

Immunohistochemical assessment of pancreatic specimens demonstrated differential NF-κBp65 expression intensities across experimental groups, directly correlating with the severity of histopathological lesions and the corresponding degree of tissue regeneration. Negative control tissue exhibited negligible nonspecific immunoreactivity for NF-κBp65 (Fig. 16a). In contrast, pancreatic specimens from the diabetic group exhibited strong positive NF-κBp65 immunoreactivity throughout the majority of islets of Langerhans and surrounding exocrine parenchyma (Fig. 16b), with concurrent islet atrophy observed in discrete regions (Fig. 16c). Conversely, animals receiving curcumin, cerium, or combination therapy demonstrated moderate NF-κBp65 immunolabeling within pancreatic islets relative to negative control baseline levels (Fig. 16d, e, f, respectively).

**Fig. 17:**
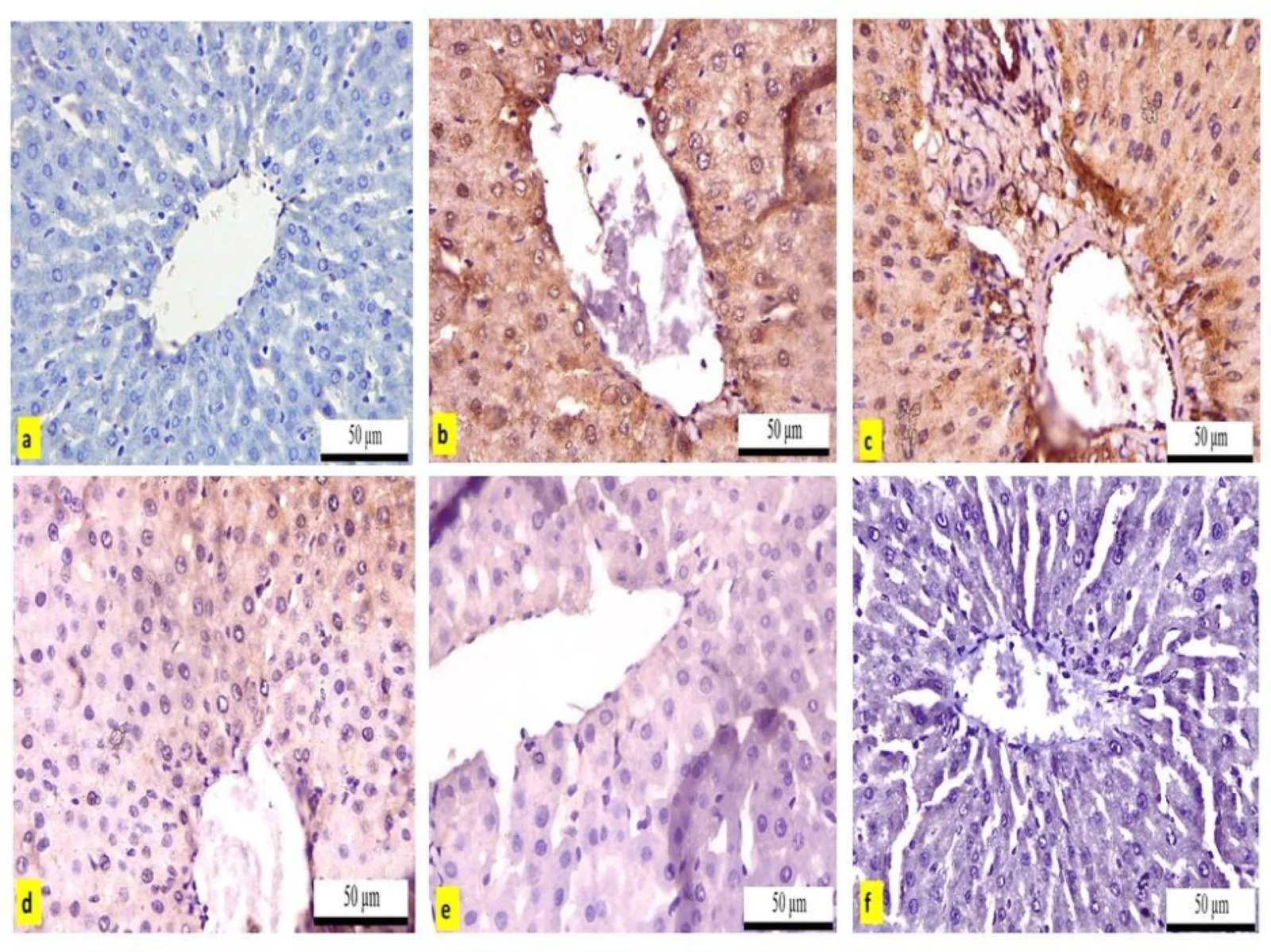
Demonstrating immune expression of NF-κBp65 stained liver sections (× 400). (**a**–**f**) (**a**) The control group exhibited a negligible NF-κBp65 immunoreaction. (**b,** **c**) The diabetic group revealed: (**b**) Intense NF-κBp65 staining in the hepatic parenchyma. (**c**) Also, there is a strong reaction at the portal area. (**d**, **e**, **f**) Curcumin & cerium, and the combination groups showed a weak expression of NF-κBp65, respectively.

Our inspection of the hepatic tissue using NF-κBp65 showed that the Negative control group had a negligible NF-κBp65 immunoreaction (Fig. 17a). In contrast, the diabetic group showed a strong NF-κBp65 staining of the hepatic parenchyma and the portal area (Fig. 17b, c). On the other hand, rats treated with curcumin and cerium or both showed a mild expression of NF-κBp65, as shown in (Fig. 17d, e, f), respectively.

##### Effect of diabetes, nanocurcumin, nanocerium, and their combination on the percent area covered by NF-ĸBp65 positive immunoreactive cells within the hepatic and pancreatic tissues of the rat. (Fig. 18)

**Fig. 18:**
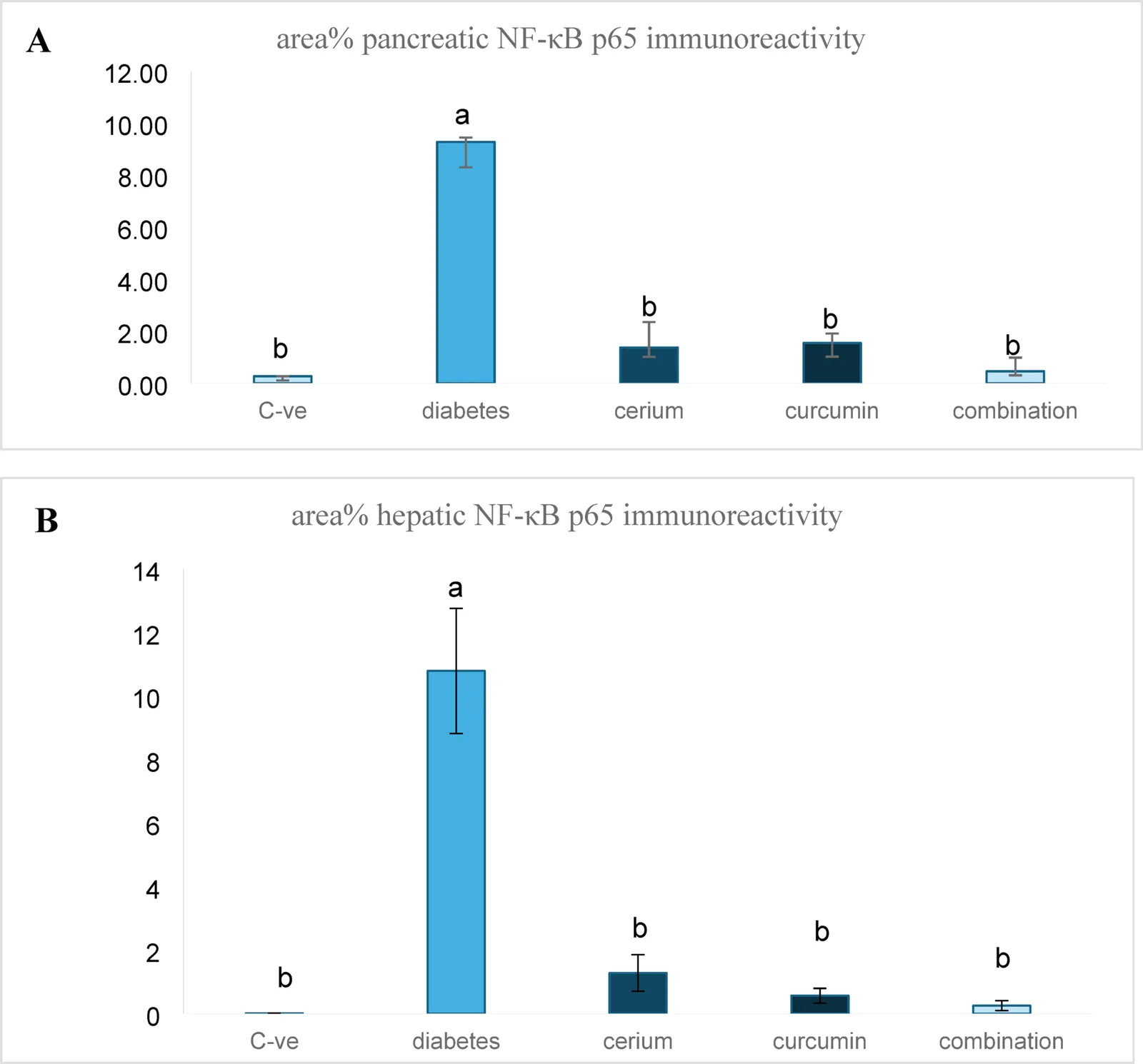
Morphometric Analysis of NF-κBp65 Immunostained Pancreatic and Hepatic Sections: Comparative Evaluation of the Protective Effects of Nanocurcumin, Nanocerium, and Their Combination Against Diabetes-Induced Pathological Changes in Rats. All data are expressed as mean ± standard error of the mean (SEM), derived from five microscopic fields per experimental group. Statistical significance was determined relative to the negative control group, with distinct superscript letters denoting significant differences at *p* < 0.05.

## Discussion

The present study offers a comprehensive evaluation of the protective potential of CNPs, Nanocurcumin, and their combinatorial formulation in alleviating systemic and cellular pathologies associated with alloxan-induced diabetes.

### Nanoparticle characterization and its relevance to this study

The therapeutic efficacy of the administered nano-formulations is intrinsically governed by their physicochemical properties^15^. In this study, high-resolution TEM characterization was prioritized to definitively confirm the core nanoscale dimensions of both the CNPs (17 ± 2 nm) and the nanocurcumin (30 ± 5 nm, spheroidal shape). This ultra-small particle size is highly relevant to our biological findings, as it guarantees a maximized surface-area-to-volume ratio, facilitating superior cellular uptake and optimal interaction with intracellular reactive oxygen species^16,43^. Furthermore, XRD analysis validated the crystalline structural integrity of the synthesized CNPs. This crystalline purity is a critical prerequisite for the distinctive auto-regenerative redox cycling that drives the potent antioxidant and anti-inflammatory outcomes observed in the hepatic and pancreatic biochemical assays^8,14^.

### Glycemic control and insulin secretion

Severe hyperglycemia coupled with hypoinsulinemia confirms the successful induction of β-cell toxicity. A salient finding of our investigation was the effect of the combined intervention, which lowered blood glucose levels by approximately 69% while augmenting serum insulin by 50%. While Nanocurcumin alone demonstrated a profound hypoglycemic effect (reducing glucose by 76%), the combination therapy appears to offer a more balanced physiological recovery. This restoration of insulin secretion suggests that the co-administration of CNPs and Nanocurcumin not only improves peripheral sensitivity but also importantly preserves the functional integrity of the pancreatic islets against oxidative destruction. These results align with those previously recorded by^4^, which demonstrated that reducing pancreatic oxidative stress via CNPs is critical for maintaining sufficient insulin biosynthesis^4^. Furthermore, it was previously reported that curcuminoids stimulate glucose uptake in peripheral tissues by modulating transporter translocation^44^, a mechanism that likely complements the insulin-sparing effects of CNPs observed in this study.

### Lipid metabolism profile

Parallel to glycemic improvements, the present study observed a significant amelioration of diabetic dyslipidemia. The diabetic state is typically characterized by an elevation in total cholesterol and triglycerides, driven by the unchecked activity of hormone-sensitive lipase in the absence of insulin. The different treatments reversed this lipotoxic profile through reducing total cholesterol and triglycerides. This anti-lipidemic effect is consistent with the findings noted that modulating insulin signaling effectively inhibits lipolysis in adipose tissue, preventing the flux of free fatty acids into the circulation^45^. By re-establishing insulin levels, the therapy mitigates the risk of cardiovascular comorbidities, a benefit previously highlighted in nanoparticle-based interventions for metabolic syndrome^46^.

### Hepatic function assessment

The protective efficacy of the nano-formulations extended to hepatocellular integrity. The diabetic group exhibited marked liver injury, evidenced by elevated serum ALT and AST activities. ALT and AST are enzymes primarily found in the liver. They are commonly used as markers to assess liver health and function. Their elevated levels often indicate liver damage or inflammation. ALT is specific for liver affections only, while AST increases in the affections of the liver, heart, muscles, kidneys, and brain^23^. Alloxan triggers the accumulation of ROS in the hepatocytes, causing an oxidative stress pathway that accounts for the elevation in hepatic toxicity markers, specifically ALT and AST^47^. The different treatments yielded a significant hepatoprotective outcome, notably by markedly reducing ALT and AST levels compared to the diabetic group. This effectively stabilizes the hepatocyte membrane against oxidative destruction. These findings are validated by^48^, who reported that Nanocurcumin significantly reduced these elevated liver enzymes, thereby confirming its ability to preserve hepatocellular function. Concomitantly,^49^ evaluated similar parameters regarding hepatic tissue damage and found consistent results with CNPs. They corroborated that these nanoparticles exert a distinct hepatoprotective effect by significantly reducing tissue damage scores and preventing injury in hepatic models, mirroring the functional recovery observed in this study.

### Renal function preservation

Similarly, the assessment of renal markers revealed a distinct nephroprotective potential. High glucose levels are inherently nephrotoxic, leading to glomerular damage reflected by increased serum urea and creatinine. The treatment regimen significantly attenuated these elevations, with creatinine levels dropping by up to 73%. This implies that the therapy effectively preserves glomerular filtration function. As previously discussed by^50^, the antioxidant properties of curcumin play a pivotal role in preventing the glycation of renal proteins, thereby delaying the onset of diabetic nephropathy^51^.

### Thyroid function profile

The evaluation of thyroid hormone homeostasis, specifically T3 and T4, revealed that the induction of diabetes did not result in significant endocrine disruption within this experimental model. Unlike the marked alterations observed in hepatic and renal parameters, serum levels of T3 and T4 in the diabetic group remained statistically comparable to those of the negative control group (*p* > 0.05). Furthermore, the administration of the therapeutic interventions, including Nanocurcumin, did not induce any marked fluctuations in thyroid hormone levels. The values recorded for the treated groups remained consistent with both the control and diabetic rats, indicating that the nano-formulations utilized in this study do not exert adverse effects on thyroid function. These findings suggest that thyroid function was preserved across all experimental conditions, a stability supported by comparable observations in recent literature regarding diabetic models and antioxidant interventions^52,53^.

### The assessment of hematological parameters

It provides critical insight into the systemic physiological impact of diabetes and the therapeutic potential of the administered nano-formulations. In the present study, the induction of diabetes resulted in distinct alterations in the erythrocytic profile, specifically a significant decline in both Hb concentrations and RBC counts compared to the negative control group. This reduction is consistent with the pathophysiology of diabetic anaemia, where oxidative stress and hyperglycemia compromise erythrocyte membrane stability and lifespan. Treatment with CNPs demonstrated the most pronounced restorative effect on the erythroid lineage, successfully normalizing both Hb and RBC levels to mirror the negative control. This suggests that the auto-regenerative antioxidant capacity of nanoceria offers preservation of erythrocyte integrity under glucotoxic stress. Conversely, the Combination therapy fully restored Hb levels but yielded an intermediate recovery in total RBC counts, while Nanocurcumin monotherapy failed to significantly improve Hb and also presented an intermediate RBC profile. Throughout these fluctuations, the MCHC remained entirely stable across all experimental groups, with no significant differences observed. This stability indicates a normochromic profile; while the absolute number of red blood cells and total hemoglobin were diminished by the diabetic state, the hemoglobin concentration within the surviving individual cells was maintained.

A unique and significant observation emerged regarding the leukocytic response. The diabetic baseline, Nanocurcumin, and CNPs groups showed no significant variation in WBC levels relative to the control. However, the administration of the combination therapy resulted in a marked elevation in WBC counts compared to all other experimental groups. This distinct spike suggests a heightened systemic leukocytic mobilization triggered specifically by the concurrent administration of the nanoparticles. While determining the precise mechanisms requires further dedicated immunological profiling, this finding highlights a differential physiological impact of the combined therapy that is absent when the agents are utilized individually. Furthermore, thrombocytic parameters exhibited specific therapeutic variances. While platelet counts in the diabetic and Nanocurcumin groups displayed overlapping statistical significance with the control baseline, both the Cerium and Combination treatments induced a significant deviation in platelet dynamics compared to the negative control. These results underscore the complex modulatory effects of these combinatorial nano-interventions on systemic hematological homeostasis^51^.

### Oxidative stress in hepatic and pancreatic tissues

A central mechanism underlying these functional improvements is the profound modulation of oxidative status. In both hepatic and pancreatic tissues, diabetes precipitated a severe redox imbalance, characterized by elevated MDA and depleted antioxidant reserves (GSH, GPx, Catalase). MDA is a toxic product of lipid peroxidation that can cause damage to cellular membranes. In contrast, GSH is a vital antioxidant that protects against oxidative stress, while catalase is an enzyme that decomposes hydrogen peroxide, thus preventing oxidative damage. GPx is an important antioxidant enzyme that plays a crucial role in protecting cells from oxidative damage. It catalyzes the reduction of hydrogen peroxide, converting it into water. This helps mitigate oxidative stress in cells^23^. Alloxan’s toxicity is mediated primarily by the generation of ROS. Alloxan interacts with intracellular thiols, such as glutathione, and undergoes a cyclic redox reaction with its reduction product, dialuric acid. This cycle generates superoxide radicals, hydrogen peroxide, and highly reactive hydroxyl radicals. These ROS cause oxidative stress, leading to the destruction of β-cells, which leads to T1D with its acute insulin deficiency. Alloxan also inhibits glucokinase, the β-cell's glucose sensor, further impairing insulin secretion, which further affects the different metabolic pathways in different organs^54^. The combined therapy acted as an effective antioxidant system. In the pancreas, the primary site of injury, it produced a remarkable increase in catalase activity. This enhanced effect likely stems from the complementary mechanisms of the agents. CNPs function as auto-regenerative radical scavengers mimicking SOD/Catalase activity^55^, while Nanocurcumin effectively suppressed lipid peroxidation (MDA) and increased antioxidant enzymes, leading to a restoration of cellular redox balance^43,48^.

### Hepatic insulin signaling gene expression

At the molecular level, the restoration of metabolic control was corroborated by changes in hepatic gene expression. The diabetic liver showed a collapsed insulin signaling pathway (reduced IR, AKT, PI3K). The combined treatment robustly reactivated this cascade, significantly upregulating IR and PI3K expression, while Nanocurcumin drove a greater than five-fold increase in AKT expression. The reactivation of the PI3K/AKT pathway is critical, as it governs hepatic glucose uptake and glycogen synthesis. This observation is supported by a similar study, which established that curcumin derivatives can bypass defective insulin receptors to directly activate downstream kinase cascades, effectively reversing hepatic insulin resistance^56^.

### Pancreatic inflammation and angiogenesis (iNOS and VEGF)

The present study elucidated the anti-inflammatory and vascular protective effects within the pancreatic microenvironment. The diabetic pancreas upregulated the mRNA expression of iNOS (indicative of nitrosative stress) and VEGF (suggestive of abnormal vascular permeability). The combined therapy demonstrated the best results in downregulating the mRNA expression of VEGF to near control levels. The different treatments significantly downregulate iNOS mRNA expression. This reduction in iNOS is particularly vital. Inhibiting inducible nitric oxide synthase prevents the accumulation of toxic nitric oxide levels that drive β-cell apoptosis^57^. Furthermore, VEGF suggests a stabilization of the islet microvasculature, preventing the intra-islet hemorrhage often seen in severe chemical diabetes models^58^.

### Histopathological study

Histopathological evaluation in the present study revealed that the diabetic state precipitated severe pancreatic injury, characterized by islet atrophy, cellular vacuolization, and ballooning degeneration, alongside the deposition of homogeneous acidophilic masses. Furthermore, the microvasculature exhibited marked capillary dilation and congestion. These degenerative alterations were effectively reversed following Nanocurcumin treatment, which significantly preserved islet morphology; these observations are in close agreement with recent findings regarding the structural benefits of Nanocurcumin^59^. Mechanistically, it is suggested that curcumin formulations protect β-cells from chemical cytotoxicity and prevent apoptotic cell death^17^. Additionally, curcumin has been shown to augment insulin secretion by directly stimulating β-cell functional activity^57^. Similarly, the administration of cerium oxide nanoparticles counteracted the pathological changes of the Islets of Langerhans. These findings are consistent with^60,61^, those previously observed that cerium nanoparticles enhanced β-cell proliferation in diabetic models. Finally, immunohistochemical analysis demonstrated that the diabetic group exhibited intense inflammatory immunoexpression when compared to controls. In contrast, treatment with Nanocurcumin and cerium nanoparticles resulted in a significant decrement in NF-κB p65 expression, confirming their ameliorative anti-inflammatory efficacy.

Additionally, histopathology examination of the liver in the diabetic group revealed severe hepatic damage, which is observed as congestion and dilatation of the central vein and blood sinusoids. Also, disorganization in the hepatic cords with numerous Von Kupffer cells. In addition, some hepatocytes exhibited ballooning and vacuolization while others displayed karyolysis and degeneration. This observation is in harmony with that clarified that the presence of diabetes mellitus contributed to the development of hepatopathy^62^. This is confirmed by a significantly strong positive NF-κBp65 immunoreactivity of hepatic tissues in the diabetic group. As observed in the recent study, treatment with Nanocurcumin effectively reduced the inflammation and treated the pathological lesions of the hepatic tissue. This is due to curcumin's anti-inflammatory, antioxidant, antimicrobial, cardioprotective, antineoplastic, neuroprotective, hepatoprotective, immune regulatory, and hypoglycemic effects^63^**.** Also, curcumin inhibited NF-κB activation^64^**.** Moreover, treatment with cerium nanoparticles ameliorated the diabetes effect on the hepatic tissue. This activity was previously reported^65^. Furthermore, the antidiabetic effects of cerium are highly significant as those of metformin, which is the standard first-line pharmacotherapeutic agent for managing type 2 diabetes mellitus^61^.

### Limitations

Although HR-TEM and XRD confirmed the crystalline structure and nanoscale dimensions of the nanomaterials, zeta potential and polydispersity index (PDI), and nanoparticle suspension stability were not evaluated. Consequently, explicit data on surface charge and colloidal stability are unavailable. To prevent aggregation, all nano-formulations were freshly prepared in PBS with 10% DMSO immediately before administration. Nonetheless, the consistent in vivo biological outcomes, including robust glycemic correction, reduced oxidative stress, and tissue preservation, demonstrate that the nanoparticles maintained sufficient functional stability, dispersion, and bioavailability to reach their physiological targets.

Also, this study is limited by the absence of hydrodynamic and surface chemistry profiling. Specifically, Dynamic Light Scattering (DLS), Polydispersity Index (PDI), and FTIR analyses were not performed. Consequently, while absolute core dimensions are established, the exact colloidal behavior and surface functionalization in physiological buffers require further elucidation. Future studies should incorporate these techniques to fully map the nanoparticles' hydrodynamic and surface profiles.

## Conclusion and future directions

This study introduces a novel dual-modality platform integrating an organic phytochemical with an auto-regenerative inorganic nanozyme to advance diabetic pharmacotherapy from glycemic management to tissue regeneration. By engineering 30 ± 5 nm spheroidal nanocurcumin, we overcome the hydrophobicity of bulk derivatives to optimize cellular permeation. Concurrently, hydrothermally synthesized 17 ± 2 nm crystalline CNPs leverage continuous Ce^3+^/Ce^4+^ redox cycling for sustained catalytic antioxidant activity. Within inflamed microenvironments, this combinatorial system neutralizes oxidative stress, ameliorates lipotoxicity, repairs pancreatic architecture, and restores endogenous insulin secretion.

This study concluded that if the primary therapeutic goal is symptomatic management (rapidly lowering circulating blood glucose and lipids), the statistical data point to Nanocurcumin as the best treatment. If the therapeutic goal is disease modification (recovering β-cell insulin secretion, neutralizing severe oxidative stress, and repairing organ architecture), the Combination is the best treatment.

## Data Availability

All data generated or analyzed during this study are included in this published article. The datasets generated and/or analyzed during the current study are available from the corresponding author on reasonable request. This study did not generate novel DNA/RNA sequences, proteomics, or macromolecular structures requiring deposition in public repositories. The accession numbers for the known gene sequences utilized for qRT-PCR primer design are provided in Table 1 of this manuscript.
